# 4+1 Gravitation in the SHP Formalism

**DOI:** 10.3390/e28040417

**Published:** 2026-04-08

**Authors:** Martin Land

**Affiliations:** Department of Computer Science, Jerusalem Multidisciplinary College, Jerusalem 91010, Israel; martin@jmc.ac.il

**Keywords:** Stueckelberg–Horwitz–Piron gravitation, ADM formalism, Hamiltonian systems, Kaluza–Klein theory

## Abstract

The Stueckelberg–Horwitz–Piron (SHP) formalism describes particles and fields traced out as spacetime events functionally dependent on an external evolution parameter τ. This approach addresses a number of difficulties associated with the problem of time. In SHP general relativity, the state of the unconstrained phase space variables $\left\{x^{\mu }\left(\tau \right),p_{\nu }\left(\tau \right)\right\}$ specifies a 4D block spacetime M(τ) that evolves to an infinitesimally close 4D block spacetime M(τ+δτ) under a scalar Hamiltonian. As the configuration of matter and energy evolves with τ it induces changes in the spacetime metric $\gamma_{\mu \nu }\left(x,\tau \right)$, leading to τ-dependent geodesic equations for the phase space variables. The 4+1 approach in gravitation generalizes the 3+1 formalism of Arnowitt, Deser, and Misner (ADM) to construct τ-dependent Einstein field equations, a canonical Hamiltonian formalism, and an initial value problem for $\gamma_{\mu \nu }\left(x,\tau \right)$. To conform to known gravitational phenomenology, we must respect the 5D symmetries associated with the free fields—the geometrical constructs relevant to M(τ) as an embedded hypersurface—and the O(3,1) symmetries of 4D matter. The 4+1 formalism has been discussed in a series of publications. The goal of this paper is to provide a systematic review of the subject, make a few corrections and some significant additions, and present the theory in a concise and orderly fashion.

## 1. Introduction

The Stueckelberg–Horwitz–Piron (SHP) formalism for classical and quantum relativistic dynamics [1,2,3,4] addresses a number of difficulties in relativity associated with the problem of time [5,6,7]. These difficulties are often attributed to the conceptual conflict between general relativity (GR) and quantum theory (see, for example, [8]), but the fundamental issue was already explored by Stueckelberg [9,10] in his foundational work on classical and quantum electrodynamics. In relativity, as in natural language, time refers to two distinct aspects of nature: instantaneous location and evolving chronology [11]. As one of the four spacetime coordinates describing the motion of a particle in a gravitational field, the time $x^{0}$ is determined by equations of motion involving derivatives of the metric. But in standard GR this time coordinate also serves as the evolution parameter by which these equations are posed and solved, either directly or through role of $x^{0}$ in the proper time. The SHP framework explicitly separates these roles by introducing an external evolution parameter τ, independent of the phase space coordinates, providing the chronological “arrow of time” by which the equations of motion may be integrated.

In a series of recent papers [12,13,14,15,16,17,18] the author has developed a 4+1 approach to 4D gravitation within the SHP formalism. In this paper, we review this work, make a few corrections and some significant additions, and present the theory in a concise and orderly fashion.

The principal goal of the 4+1 approach is to formulate generalized field equations for a local 4D spacetime metric $\gamma_{\mu \nu }\left(x,\tau \right)$ that evolves under the influence of matter and field sources $T_{\mu \nu }\left(x,\tau \right)$, themselves evolving as spacetime events $x^{\mu }\left(\tau \right)$ trace out worldline trajectories under the monotonic advance of τ. As we shall see, attention to the symmetries required for such field equations leads to a 4+1 formalism analogous to the 3+1 geometrodynamics of Arnowitt, Deser, and Misner (ADM) [19]. The resulting formalism recovers standard Einstein GR in the equilibrium limit of no τ-evolution and is compatible with standard Kaluza–Klein theory in this limit. Following a brief review of the SHP formalism, motivating the 4+1 approach, we will discuss the geodesic equations in this framework and develop the field theory for the metric.

### 1.1. Hamiltonian Structure on 8D Phase Space

In 1937, Fock [20] generalized the phase space of nonrelativistic classical mechanics to 4D Minkowski spacetime as

(1) $$\left\{x_{n}^{i}\left(t\right),p_{i}^{n}\left(t\right)\right\},\;i=1,\cdots ,3\;⟶\;\left\{x_{n}^{\mu }\left(s\right),p_{\mu }^{n}\left(s\right)\right\},\;\mu =0,\cdots ,3\;$$

where n=1,⋯,N. Here, $x_{n}^{0}\left(s\right)=ct_{n}\left(s\right)$ represents the time registered on a laboratory clock, and Fock identified the monotonically advancing scalar parameter *s* with the proper time of the motion. Writing the velocity

(2) $${\dot{x}}^{\mu }\left(s\right)=\frac{dx^{\mu }}{ds}$$

and the Lorentz scalar action

(3) $$S=\int ds\;L\left(x,\dot{x},s\right)=\int ds\left(\frac{1}{2}M{\dot{x}}^{\mu }{\dot{x}}_{\mu }+\frac{e}{c}{\dot{x}}_{\mu }A^{\mu }\right)$$

he showed that the covariant Euler–Lagrange equations

(4) $$\frac{d}{ds}\frac{\partial L}{\partial {\dot{x}}^{\mu }}-\frac{\partial L}{\partial x^{\mu }}=0$$

lead to the classical Lorentz force in Maxwell electrodynamics.

In 1941, Stueckelberg [9,10] proposed that a particle–antiparticle interaction could be described by a single evolving event interacting with an electromagnetic field in such a way that its energy becomes negative and it reverses direction in coordinate time $x^{0}$. This description, central to quantum field theory, became known as the Feynman–Stueckelberg formalism [21,22]. But Stueckelberg observed that for classical worldlines, the evolution parameter cannot be identified with the proper time. While crossing the spacelike region that separates future-oriented trajectories from past-oriented trajectories, the sign of ${\dot{x}}^{2}$ will change twice, and so the computed proper time interval

(5) $$\frac{1}{c}\sqrt{-\eta_{\mu \nu }dx^{\mu }dx^{\nu }}=\frac{1}{c}\sqrt{-{\dot{x}}^{2}\left(s\right)}\;ds\;\;\eta_{\mu \nu }=\mathrm{diag}\left(-1,1,1,1\right)$$

cannot provide a consistent parameterization. Therefore, Stueckelberg argued for the introduction of an external evolution parameter τ, analogous to the Newtonian time *t* and related to the proper time *s* through the dynamical relation $c^{2}ds^{2}\left(\tau \right)=-{\dot{x}}^{2}\left(\tau \right)d\tau^{2}$. Implicit in the form of Fock’s action (3) is that ${\dot{x}}^{2}$ is not *a priori* constrained, but it is nevertheless conserved under the electromagnetic interaction as

(6) $$M{\ddot{x}}^{\mu }=eF^{\mu \nu }{\dot{x}}_{\nu }\;⟶\;\frac{d}{d\tau }{\dot{x}}^{2}=\frac{e}{M}F^{\mu \nu }{\dot{x}}_{\mu }{\dot{x}}_{\nu }=0.$$

To overcome this restriction, Stueckelberg proposed a modified Lorentz force that included a new vector field, but found no satisfying way to justify its presence in classical relativistic mechanics.

In 1973, Horwitz and Piron [23] advanced Stueckelberg’s work, constructing a manifestly covariant relativistic mechanics with interactions on an unconstrained phase space, effectively demoting constraints associated with the presumed dynamics of the status of conservation laws. For example, one may introduce a scalar potential V(x) to the action (3), producing the modified Lorentz force

(7) $$M{\ddot{x}}^{\mu }=eF^{\mu \nu }{\dot{x}}_{\nu }-\frac{\partial V}{\partial x_{\mu }}$$

sought by Stueckelberg. More generally, by making the potentials τ-dependent and introducing a scalar gauge field $a_{5}\left(x,\tau \right)$,

(8) $$S_{\mathrm{SHP}}=\int d\tau \;\frac{1}{2}M{\dot{x}}^{\mu }{\dot{x}}_{\mu }+\frac{e}{c}{\dot{x}}^{\mu }a_{\mu }\left(x,\tau \right)+\frac{e}{c}c_{5}a_{5}\left(x,\tau \right)$$

the Fock action is made maximally U(1) gauge-invariant [3,24] as

(9) $$a_{\mu }\;⟶\;a_{\mu }+\partial_{\mu }\Lambda \left(x,\tau \right)\;a_{5}\;⟶\;a_{5}+\frac{1}{c_{5}}\partial_{\tau }\Lambda \left(x,\tau \right)$$

where we introduce a constant fifth velocity $c_{5}$ to write $x^{5}=c_{5}\tau$ in analogy to the notation $x^{0}=ct$. Partitioning the Greek indices as

(10) α,β,γ,δ=0,1,2,3,5λ,μ,ν,ρ…=0,1,2,3

we see that the O(3,1) scalars ${\dot{x}}^{\mu }{\dot{x}}_{\mu }$, ${\dot{x}}^{\mu }a_{\mu }$, and $a_{5}$ leave the action

(11) $$S_{\mathrm{SHP}}=\int d\tau \;\frac{1}{2}M{\dot{x}}^{\mu }{\dot{x}}_{\mu }+\frac{e}{c}{\dot{x}}^{\beta }a_{\beta }\left(x,\tau \right)$$

4D Lorentz-invariant, despite the 5D gauge invariance $a_{\alpha }\left(x,\tau \right)⟶a_{\alpha }\left(x,\tau \right)+\partial_{\alpha }\Lambda \left(x,\tau \right)$.

The canonical momentum is

(12) $$p_{\mu }=\frac{\partial L}{\partial {\dot{x}}^{\mu }}=M{\dot{x}}_{\mu }+\frac{e}{c}a_{\mu }$$

leading to the O(3,1) scalar Hamiltonian

(13) $$K=p_{\mu }{\dot{x}}^{\mu }-L=\frac{1}{2M}\left(p^{\mu }-\frac{e}{c}a^{\mu }\right)\left(p_{\mu }-\frac{e}{c}a_{\mu }\right)-\frac{e}{c}c_{5}a_{5}$$

subject to the canonical equations of motion

(14) $${\dot{x}}^{\mu }=\frac{dx^{\mu }}{d\tau }=\frac{\partial K}{\partial p_{\mu }}\;\;\;{\dot{p}}_{\mu }=\frac{dp_{\mu }}{d\tau }=-\frac{\partial K}{\partial x^{\mu }}$$

and 4D Poisson bracket structure. The Stueckelberg–Schrodinger equation

(15) $$i\hbar \partial_{\tau }\psi \left(x,\tau \right)=\left[\frac{1}{2M}\left(p^{\mu }-\frac{e}{c}a^{\mu }\right)\left(p_{\mu }-\frac{e}{c}a_{\mu }\right)-\frac{e}{c}c_{5}a_{5}\right]\psi \left(x,\tau \right)$$

provides a covariant quantum mechanics with relativistic generalizations to the standard central force problems [3,4].

### 1.2. Motivation for a τ-Dependent Local Metric

In the Stueckelberg–Horwitz–Piron (SHP) formalism, particle worldlines are traced out by the evolution of classical events $x^{\mu }\left(\tau \right)$ or quantum events ψ(x,τ) as the external evolution parameter τ advances monotonically. Even a ‘static’ classical particle in its rest frame is characterized by its uniform evolution along its *t*-axis as

(16) $$x\left(\tau \right)=\left(x^{0}\left(\tau \right),x\left(\tau \right)\right)=\left(c\tau ,0\right)$$

and event trajectories carrying electromagnetic charge induce five τ-dependent gauge fields $a_{\alpha }\left(x,\tau \right)$. In curved spacetime, these worldlines and fields produce a scalar event density ρ(x,τ) and an energy–momentum density $T_{\mu \nu }\left(x,\tau \right)$ that evolve with τ and act as sources for the gravitational field. Following Wheeler’s description of geometrodynamics, “spacetime tells matter how to move; matter tells spacetime how to curve” [25], the τ-evolution of the mass/energy/momentum distribution associated with these events entails the τ-evolution of spacetime curvature described by a local metric $\gamma_{\mu \nu }\left(x,\tau \right)$. In the presence of τ-dependent matter and fields, a τ-independent 4D metric would have the character of an absolute background field, contradicting the goals of general relativity. Evolution of the metric with τ naturally leads to generalized geodesic equations and requires generalized field equations.

General relativity (GR) begins with consideration of the squared interval

(17) $$\delta x^{2}=\gamma_{\mu \nu }\delta x^{\mu }\delta x^{\nu }={\left(x_{2}-x_{1}\right)}^{2}$$

between two neighboring points of the spacetime manifold M viewed as a 4D block universe. The spacetime trajectory of a material event is a continuous sequence of events $x^{\mu }\left(s\right)$ of timelike separation in M, so that *s* may be the proper time. Writing the invariant interval (17) as

(18) $$\delta x^{2}=\gamma_{\mu \nu }\delta x^{\mu }\delta x^{\nu }=\gamma_{\mu \nu }\frac{dx^{\mu }}{ds}\frac{dx^{\nu }}{ds}\delta s^{2}=\gamma_{\mu \nu }{\dot{x}}^{\mu }{\dot{x}}^{\nu }\delta s^{2}$$

suggests a dynamical description of the trajectory by the action

(19) $$S=\int ds\;\frac{1}{2}\;\gamma_{\mu \nu }{\dot{x}}^{\mu }{\dot{x}}^{\nu }$$

on which the constraint ${\dot{x}}^{2}=-c^{2}$ may be imposed *a posteriori*.

In the SHP framework, a physical event $x^{\mu }\left(\tau \right)$ is an irreversible occurrence at τ with spacetime coordinates $x^{\mu }$. Thus, the 4D block universe M(τ) similarly occurs at τ, representing the instantaneous manifold of general relativity. The scalar Hamiltonian *K* generates evolution of M(τ) to an infinitesimally close 4D block universe M(τ+dτ) occurring at τ+dτ. We may therefore consider the interval

(20) $$x_{2}\left(\tau +\delta \tau \right)-x_{1}\left(\tau \right)≃x_{2}\left(\tau \right)+\frac{dx(\tau )}{d\tau }\delta \tau -x_{1}\left(\tau \right)=\delta x+\frac{dx(\tau )}{d\tau }\delta \tau$$

which introduces a notion of 5D distance that combines the *geometrical distance* δx between any two arbitrary points in M(τ) and the *dynamical distance* between events generated by the Hamiltonian that evolves M(τ)⟶M(τ+δτ).

We may regard the event $X=\left(x,x^{5}\right)=\left(x^{0},\ldots ,x^{3},c_{5}\tau \right)$ as a point in a pseudo-spacetime $M_{5}$ obtained as the image of an injective mapping

(21) $$\Phi :M\;⟶\;M_{5}=M\times R\;\;\;X=\Phi \left(x,\tau \right)=\left(x,c_{5}\tau \right).$$

For two neighboring points $X_{1},X_{2}\in M_{5}$ we may write

(22) $$X_{1}=\left(x_{1}\left(\tau \right),c_{5}\tau \right)\;\;X_{2}=\left(x_{2}\left(\tau +\delta \tau \right),c_{5}\left(\tau +\delta \tau \right)\right)$$

so that

(23) $$\delta X=X_{2}-X_{1}=\left(\delta x+\frac{dx}{d\tau }\delta \tau ,c_{5}\delta \tau \right)$$

suggests a 5D invariant interval of the form

(24) $$\delta X^{2}=\gamma_{\mu \nu }\left(\delta x^{\mu }+\frac{dx^{\mu }}{d\tau }\delta \tau \right)\left(\delta x^{\nu }+\frac{dx^{\nu }}{d\tau }\delta \tau \right)+\sigma c_{5}^{2}\delta \tau^{2}=g_{\alpha \beta }\left(x,\tau \right)\delta X^{\alpha }\delta X^{\beta }$$

where σ=±1. This squared interval leads to the general Lagrangian

(25) $$L=\frac{1}{2}Mg_{\alpha \beta }\left(x^{\mu },x^{5}\right){\dot{X}}^{\alpha }{\dot{X}}^{\beta }\;\mu =0,1,2,3\;\alpha ,\beta =0,1,2,3,5$$

from which we may find equations of motion in the manifold determined by some local metric $g_{\alpha \beta }$. This structure permits us to characterize 4D spacetime M as a hypersurface embedded in $M_{5}$. The natural foliation of $M_{5}$ into equal-τ spacetimes M(τ) is then analogous to the foliation of 4D spacetime into equal-*t* spacelike hypersurfaces in standard GR. As shown in Section 3, this facilitates the generalization of 3+1 geometrodynamics to a 4+1 formalism for the field equations describing the induced 4D metric $\gamma_{\mu \nu }\left(x,\tau \right)$.

### 1.3. Overview of Paper

The remainder of this paper is structured as follows. In Section 2 we find the equations of motion from a Lagrangian of the form (25), noting the requirement to preserve the 4D symmetry associated with particle motion. Section 3 presents the generalized Einstein field equations for the τ-dependent local metric. Once again, these equations must respect the 5D symmetries of free fields as geometric objects, while respecting the 4D symmetries of the matter terms. In Section 4 we generalize the 3+1 formalism [19] developed by Arnowitt, Deser, and Misner (ADM) to 4+1. In this approach we decompose the generalized field equations into an initial value problem, posed as first-order evolution equations for the induced 4D metric $\gamma_{\mu \nu }\left(x,\tau \right)$ along with the extrinsic curvature $K_{\mu \nu }\left(x,\tau \right)$. Section 5 presents the 4+1 formalism as a canonical Hamiltonian theory. As in ADM, strategic combinations of $\gamma_{\mu \nu }\left(x,\tau \right)$ and $K_{\mu \nu }\left(x,\tau \right)$ lead to a canonical Hamiltonian formalism for SHP GR derived from a scalar action. Section 6 discusses the perturbative theory for weak gravitation and shows that in this approximation the initial value problem can be obtained directly from the 5D Ricci tensor. In Section 7 we compare this 4+1 formalism to the Kaluza–Klein model [26,27] for 4D gravitation and electromagnetism and establish the compatibility of the two approaches only in τ-independent equilibrium. Section 8 discusses some examples and some prospects for gravitation in the SHP formalism.

### 1.4. Revision of Earlier Work

This paper revises the approach in earlier work on SHP gravitation, corrects a few significant errors, and introduces a few innovations. The presentation in [12] recognized the importance of preserving the 4D symmetry of particle motions, but attempted to impose this restriction through modification of the connection $\Gamma_{\alpha \beta }^{\gamma }$. This approach was not entirely successful and so was replaced in [13] by a generalization of the ADM 3+1 formalism to 4+1. In [14] the 4+1 formalism was applied to the linearized equations for weak gravitation, and it was shown that in this approximation the initial value problem can be obtained directly from the 5D Ricci tensor. However in both [13,14] the symmetry breaking for the matter terms was incorrectly handled, leading to small but significant errors in the equations for the initial value problem. The symmetry breaking was corrected for weak gravitation in [15] but this paper did not yet provide a formulation of symmetry breaking for the full nonlinear field equations. A correct formulation of the full field equations was presented in [16] by introducing the quintrad frame with a vielbein field, so that the method of symmetry breaking in weak gravitation could be applied to the general metric. In [17] an expression for an evolving metric was obtained in weak gravitation using an approximate Green’s function for the 5D wave equation. The difficulties associated with this metric were demonstrated and traced back to the approximation applied through the Green’s function. In [18] an ansatz for the desired metric induced by an event evolving uniformly along its $x^{0}$-axis in its rest frame was introduced. While this metric is indeed no more than an ansatz, it demonstrates many of the expected properties of a metric in the 4+1 formalism. And as with any ansatz, this form is a starting point for exploration of SHP gravitation and its implications.

As indicated in Section 1.3, this paper presents the 4+1 theory in a revised and more orderly form than the meandering path it took in its initial formulation. Here, the quintrad frame and its 4+1 foliation are introduced from the start, so that the symmetry-broken 5D field equations can be formulated before jumping into their decomposition in evolving 4D quantities. This decomposition then follows the generalization of 3+1 ADM as previously published, but using the revised 5D gravitational field equation, leading to a correct and consistent initial value problem. Although these changes do not appear explicitly in the equations for the ADM Hamiltonian formulation, they will be present in any calculation. With the corrected initial value problem in place, the weak gravitation approximation is then presented, obtaining the evolution equations and constraints in correct form. Here, the discussion of equilibrium has been expanded from earlier work, using the corrected evolution equation to describe the possibility of a nonzero mass density in the form of an additional “dark” source term. Finally, the comparison of 4+1 SHP gravitation with the 4+1 Kaluza–Klein theory is entirely new.

## 2. SHP Particle Dynamics

Varying the scalar event Lagrangian

(26) $$L=\frac{1}{2}Mg_{\alpha \beta }{\dot{X}}^{\alpha }{\dot{X}}^{\beta }\;\;X^{\alpha }\in M_{5}\;\alpha ,\beta ,\gamma =0,1,2,3,5$$

with respect to $X^{\gamma }$ leads to 5D geodesic equations

(27) $$\frac{D{\dot{X}}^{\gamma }}{D\tau }={\ddot{X}}^{\gamma }+\Gamma_{\alpha \beta }^{\gamma }{\dot{X}}^{\alpha }{\dot{X}}^{\beta }=0\;\;\Gamma_{\beta \gamma }^{\alpha }=\frac{1}{2}g^{\alpha \delta }\left(\frac{\partial g_{\delta \beta }}{\partial X^{\gamma }}+\frac{\partial g_{\delta \gamma }}{\partial X^{\beta }}-\frac{\partial g_{\beta \gamma }}{\partial X^{\delta }}\right)$$

with 5D Christoffel symbols in standard form. The apparent 5D symmetry of these equations is broken to O(3,1) by imposing ${\dot{x}}^{5}=c_{5}\;⟶\;{\ddot{x}}^{5}=0$ as a constraint, so that

(28) $$\begin{array}{c} \frac{D{\dot{x}}^{\mu }}{D\tau }={\ddot{x}}^{\mu }+\Gamma_{\alpha \beta }^{\mu }{\dot{X}}^{\alpha }{\dot{X}}^{\beta }={\ddot{x}}^{\mu }+\Gamma_{\nu \sigma }^{\mu }{\dot{x}}^{\nu }{\dot{x}}^{\sigma }+2c_{5}\Gamma_{5\nu }^{\mu }{\dot{x}}^{\nu }+c_{5}^{2}\Gamma_{55}^{\mu }=0 \end{array}$$

(29) $$\begin{array}{c} \frac{D{\dot{x}}^{5}}{D\tau }={\ddot{x}}^{5}\equiv 0 \end{array}$$

describe the dynamical degrees of freedom. In case $g_{5\alpha }=0$ and $\partial_{\tau }g_{\mu \nu }=0$, the connection components $\Gamma_{5\alpha }^{\mu }$ vanish, recovering the standard 4D GR geodesic equations. Writing the canonical momentum

(30) $$p_{\mu }=\frac{\partial L}{\partial {\dot{x}}^{\mu }}=M\left(g_{\mu \nu }{\dot{x}}^{\nu }+c_{5}g_{\mu 5}\right)$$

the Legendre transformation produces the Hamiltonian

(31) $$K=\frac{1}{2M}p^{2}+\frac{1}{2}c_{5}g_{55}g^{5\mu }p_{\mu }-\frac{1}{2}c_{5}g_{5\mu }g^{\mu \lambda }p_{\lambda }+\frac{1}{2}Mc_{5}^{2}g_{5\mu }g^{\mu \lambda }g_{\lambda 5}+\frac{1}{2}Mc_{5}^{2}g_{55}$$

representing the total 5D mass of the motion. It is useful here to think of mass as arising from $p^{2}=M^{2}\;{\dot{x}}^{2}$, which is not constrained to be a constant of the motion. Thus, particle energy may change with no corresponding change in 3-momentum, producing a change in the mass $p^{2}$ associated with the trajectory.

For $g^{5\mu }=0$ the Hamiltonian takes the simple form

(32) $$K=\frac{1}{2M}p^{\mu }p_{\mu }+\frac{1}{2}Mc_{5}^{2}\;g_{55}$$

with $g_{55}\left(x,\tau \right)$ appearing as a τ-dependent scalar potential on 4D spacetime. While such a potential would be non-geodesic in standard GR, it follows simply from the 5D geodesic equations. The Hamilton Equations (14) permit us to write the Poisson bracket relations

(33) $$\left\{F,G\right\}=\frac{\partial F}{\partial x^{\alpha }}\frac{\partial G}{\partial p_{\alpha }}-\frac{\partial F}{\partial p_{\alpha }}\frac{\partial G}{\partial x^{\alpha }}=\frac{\partial F}{\partial x^{\mu }}\frac{\partial G}{\partial p_{\mu }}-\frac{\partial F}{\partial p_{\mu }}\frac{\partial G}{\partial x^{\mu }}$$

where the second equality follows from $p_{5}\equiv 0$. Since

(34) $$\frac{dF}{d\tau }=\left\{F,K\right\}+\frac{\partial F}{\partial \tau }$$

for any scalar function *F* on phase space, the Hamiltonian is conserved unless it depends explicitly on τ through $g_{\alpha \beta }\left(x,\tau \right)$. Nevertheless, the mass $m=-p^{\mu }p_{\mu }/2M$ associated with the event trajectory may vary under $g_{5\alpha }$. For a discussion of geodesic equations and quantum mechanics with a τ-independent local metric, see [28,29].

In the Newtonian limit, neglecting ${\dot{x}}^{i}/c<1$ for i=1,2,3 and taking $g_{\alpha \beta }$ to be *t*-independent, the equations of motion reduce to

(35) $$\frac{d^{2}t}{d\tau^{2}}=\frac{dt}{d\tau }\;\partial_{\tau }g_{00}\;\;\ddot{x}=\frac{1}{2}c^{2}{\left(\frac{dt}{d\tau }\right)}^{2}\nabla g_{00}+\frac{1}{2}c_{5}^{2}\nabla g_{55}$$

which deviate from the standard nonrelativistic case if $g_{00}$ is τ-dependent, producing an acceleration in the time coordinate *t*. A simple post-Newtonian expression is found by setting $\nabla g_{55}=0$ and introducing an additive perturbation δM(τ) to the mass parameter *M* in the Schwarzschild metric $g_{00}=-1+2GM/c^{2}R$. The *t* equation admits the solution

(36) $$\frac{dt}{d\tau }=\exp\left(\frac{2G}{c^{2}R}\;\delta M\right)$$

which recovers $\ddot{t}=0$ in the absence of the perturbation. In spherical coordinates, angular momentum $L=MR^{2}\dot{\phi }$ is conserved and the radial equation is

(37) $$\ddot{R}-\frac{L^{2}}{M^{2}R^{3}}+\exp\left(\frac{4G}{c^{2}R}\;\delta M\right)\frac{GM_{0}}{R^{2}}=0,$$

which becomes Newtonian gravitation for δM=0. We find the Hamiltonian for the test event in this coordinate system to be

(38) $$K=\frac{1}{2}Mg_{\alpha \beta }{\dot{X}}^{\alpha }{\dot{X}}^{\beta }=-\frac{1}{2}Mc^{2}\left(1-\frac{2GM_{0}}{c^{2}R}\right)\exp\left(\frac{4G}{c^{2}R}\;\delta M\right)+\frac{1}{2}M{\dot{R}}^{2}+\frac{1}{2}\frac{L^{2}}{MR^{2}}$$

with τ derivative

(39) $$\frac{d}{d\tau }K=\exp\left(\frac{4G}{c^{2}R}\;\delta M\right)\left(-\frac{GM}{r}+\frac{4G^{2}MM_{0}}{c^{2}R^{2}}\right)\frac{d}{d\tau }\delta M$$

so that the total mass *K* is not conserved in the presence of a variable-mass gravitational source.

The scalar event density ρ(x,τ) is defined as the number of events per spacetime volume. The 5-component event current is

(40) $$j^{\alpha }\left(x,\tau \right)=M\rho \left(x,\tau \right){\dot{x}}^{\alpha }\left(\tau \right)$$

where $j^{5}=Mc_{5}\;\rho \left(x,\tau \right)$ is an O(3,1) scalar and not the 5-component of a vector. Therefore, the continuity equation is

(41) $$\nabla_{\alpha }j^{\alpha }=\frac{\partial j^{\alpha }}{\partial x^{\alpha }}+j^{\gamma }\Gamma_{\gamma \alpha }^{\alpha }=\frac{\partial \rho }{\partial \tau }+\nabla_{\mu }j^{\mu }=0$$

and by virtue of the continuity and geodesic equations, the mass–energy–momentum tensor

(42) $$T^{\alpha \beta }=M\rho {\dot{x}}^{\alpha }{\dot{x}}^{\beta }⟶\left\{\begin{array}{c} T^{\mu \nu }=M\rho {\dot{x}}^{\mu }{\dot{x}}^{\nu },\\ T^{5\beta }={\dot{x}}^{5}{\dot{x}}^{\beta }M\rho =c_{5}j^{\beta } \end{array}\right.$$

is conserved as

(43) $$\nabla_{\alpha }T^{\alpha \beta }=0$$

which as in (41) implies only O(3,1) symmetry in the contracted index α. As shown in [30] the spacetime components of $T_{\mu \nu }$ have the usual interpretation, with $T_{00}$ being the energy flux and $T_{0i}$ a 3-momentum flux for i=1,2,3, while $T_{55}$ is the mass flux and $T_{5\mu }$ is a 4-momentum flux for μ=0,1,2,3. Varying the electromagnetic action (11) with respect to $x^{\mu }$ provides the Lorentz force

(44) $$\begin{array}{c} M{\ddot{x}}^{\mu }\left(\tau \right)=\frac{e}{c}\left[f_{\;\;\nu }^{\mu }\left(x,\tau \right){\dot{x}}^{\nu }\left(\tau \right)+c_{5}f_{\;\;5}^{\mu }\left(x,\tau \right)\right]=\frac{e}{c}f_{\;\;\alpha }^{\mu }\left(x,\tau \right){\dot{x}}^{\alpha }\left(\tau \right)\\ \frac{d}{d\tau }\left(-\frac{1}{2}M{\dot{x}}^{2}\right)=-M{\dot{x}}^{\mu }{\ddot{x}}_{\mu }=\frac{ec_{5}}{c}f_{5\mu }{\dot{x}}^{\mu } \end{array}$$

where

(45) $$f_{\alpha \beta }=\partial_{\alpha }a_{\beta }-\partial_{\beta }a_{\alpha }$$

is the field strength. We see that $f_{5\mu }$ can change the mass $M{\dot{x}}^{2}$ of an evolving matter event, as anticipated by Stueckelberg. The mass–energy–momentum tensor for the electromagnetic field satisfies [24,30]

(46) $$\partial_{\alpha }T_{field}^{\alpha \beta }=-\frac{e}{c}f^{\beta \alpha }{\dot{x}}_{\alpha }\left(\tau \right)\delta \left(x-x\left(\tau \right)\right)\;\;T_{field}^{\alpha \beta }=\frac{1}{c}\left(f^{\alpha \gamma }f_{\;\;\gamma }^{\beta }-\frac{1}{4}\eta^{\alpha \beta }f^{\delta \varepsilon }f_{\delta \varepsilon }\right)$$

which together with the Lorentz force (44) leads to

(47) $$\frac{d}{d\tau }\left[\int d^{4}x\;T_{field}^{55}+\eta^{55}\left(-\frac{1}{2}M{\dot{x}}^{2}\right)\right]=0$$

expressing conservation of the combined mass of particles and fields. In these expressions, the scalar $T_{field}^{55}$ represents the spacetime density of mass associated with the fields, in analogy to the energy density $T_{field}^{00}$. Similarly, $T_{field}^{5\mu }$ is a Poynting 4-vector describing the flow of electromagnetic 4-momentum into spacetime, in analogy with the standard Poynting vector $T_{field}^{0i}$.

Since the 5D Ricci tensor, obtained in the usual manner from the metric $g_{\alpha \beta }\left(x,\tau \right)$, is invariant under translations $x^{\prime \alpha }=x^{\alpha }+\Lambda^{\alpha }\left(x,\tau \right)$, the Bianchi identity [31]

(48) $$\nabla_{\alpha }\left(R^{\alpha \beta }-\frac{1}{2}g^{\alpha \beta }R\right)=0$$

must hold. In the absence of the matter current, the first of (46) provides

(49) $$\nabla_{\alpha }T_{field}^{\alpha \beta }=0$$

and so together with (43) we may write 5D Einstein equations

(50) $$R_{\alpha \beta }-\frac{1}{2}g_{\alpha \beta }R=\frac{8\pi G}{c^{4}}\left(T_{\alpha \beta }+T_{\alpha \beta }^{field}\right)$$

with the mass–energy–momentum tensors as sources. Nevertheless, the spacetime symmetry of $T^{\alpha \beta }$ should be no larger than O(3,1) as a matter of principle, and so the apparent 5D symmetry of (50) must be broken to 4+1. This symmetry breaking is most conveniently accomplished in a quintrad frame.

## 3. SHP Field Equations

### 3.1. Quintrad Frame

The tangent space $T\left(M_{5}\right)$ of the pseudo-spacetime $M_{5}$ can be given the standard coordinate basis

(51) $$g_{\alpha }=\partial_{\alpha }=\frac{\partial }{\partial X^{\alpha }}$$

while the dual space $T^{*}\left(M_{5}\right)$ has the basis 1-forms

(52) $$g^{\alpha }=dX^{\alpha }$$

which satisfy

(53) $$g^{\alpha }\left(g_{\beta }\right)=g^{\alpha }\cdot g_{\beta }=\delta_{\beta }^{\alpha }\;\;g_{\alpha }\cdot g_{\beta }=g_{\alpha \beta }\;\;g^{\alpha }\cdot g^{\beta }=g^{\alpha \beta }.$$

Extending the vierbein formalism [32] to 5D we define the constant quintrad frames $\left\{e_{a}\right\}$ for $T\left(M_{5}\right)$ and $\left\{e^{a}\right\}$ for $T^{*}\left(M_{5}\right)$, which satisfy

(54) $$e_{a}\cdot e_{b}=\eta_{ab}\;\;e^{a}\cdot e^{b}=\eta^{ab}\;\;\partial_{a}e_{b}=\partial_{a}e^{b}=0.$$

By convention the Latin letters

(55) $$a,b,c,\cdots =0,1,2,3,5\;\;\eta_{ab}=\mathrm{diag}\left(-1,1,1,1,\sigma \right)$$

indicate reference to the quintrad, where $\sigma =\eta_{55}=\eta^{55}=\pm 1$ provides a metric pseudo-signature on $M_{5}$. The position-dependent coordinate bases are related to the quintrad through the vielbein field

(56) $$g_{\alpha }=E_{\alpha }^{\;a}\left(X\right)e_{a}\;\;g^{\alpha }={\bar{E}}_{\;a}^{\alpha }\left(X\right)e^{a}$$

which is invertible as

(57) $$e_{a}=e_{\;a}^{\alpha }\left(X\right)g_{\alpha }\;\;e^{a}={\bar{e}}_{\alpha }^{\;a}\left(X\right)g^{\alpha }.$$

Consistency requires that

(58) $$e_{\;a}^{\alpha }\left(X\right)E_{\beta }^{\;a}\left(X\right)=\delta_{\beta }^{\alpha }\;\;e_{\;a}^{\alpha }\left(X\right)E_{\alpha }^{\;b}\left(X\right)=\delta_{a}^{b}$$

with similar relations for ${\bar{E}}_{\;a}^{\alpha }$ and ${\bar{e}}_{\alpha }^{\;a}$. Duality imposes the conditions

(59) $$\begin{array}{c} \delta_{a^{\prime }}^{a}=e^{a}\cdot e_{a^{\prime }}={\bar{e}}_{\alpha }^{\;a}g^{\alpha }\cdot e_{\;a^{\prime }}^{\beta }g_{\beta }={\bar{e}}_{\alpha }^{\;a}e_{\;a^{\prime }}^{\beta }\delta_{\beta }^{\alpha }={\bar{e}}_{\alpha }^{\;a}e_{\;a^{\prime }}^{\alpha }\\ \delta_{\alpha^{\prime }}^{\alpha }=g^{\alpha }\cdot g_{\alpha^{\prime }}={\bar{E}}_{\;a}^{\alpha }e^{a}\cdot E_{\alpha^{\prime }}^{\;b}e_{b}={\bar{E}}_{\;a}^{\alpha }E_{\alpha^{\prime }}^{\;b}\delta_{b}^{a}={\bar{E}}_{\;a}^{\alpha }E_{\alpha^{\prime }}^{\;a} \end{array}$$

which together with (58) put the transformation equations into the form

(60) $$\begin{array}{ccc} g_{\alpha }=E_{\alpha }^{\;a}e_{a} & \;\;\; & e_{a}=e_{\;a}^{\alpha }g_{\alpha }\\ g^{\alpha }=e_{\;a}^{\alpha }e^{a} & \;\; & e^{a}=E_{\alpha }^{\;a}g^{\alpha }. \end{array}$$

The vielbein field thus induces the local metric through

(61) $$\begin{array}{ccc} g_{\alpha \beta }=g_{\alpha }\cdot g_{\beta }=\eta_{ab}\;E_{\alpha }^{\;a}E_{\beta }^{\;b} & \;\; & g^{\alpha \beta }=g^{\alpha }\cdot g^{\beta }=\eta^{ab}\;e_{\;a}^{\alpha }e_{\;b}^{\beta }\\ \eta_{ab}=e_{a}\cdot e_{b}=g_{\alpha \beta }e_{\;a}^{\alpha }e_{\;b}^{\beta } & \;\; & \eta^{ab}=e^{a}\cdot e^{b}=g^{\alpha \beta }E_{\alpha }^{\;a}E_{\beta }^{\;b} \end{array}$$

where we use (56) and (57). Since any vector in $T\left(M_{5}\right)$ can be written

(62) $$V=V^{\alpha }g_{\alpha }=\left[V^{\alpha }E_{\alpha }^{\;a}\left(X\right)\right]e_{a}=V^{a}e_{a}$$

we may view

(63) $$X_{b}^{a}=E_{\alpha }^{\;a}e_{a}^{\;\beta }X_{\beta }^{\alpha }\;\;\;X_{\beta }^{\alpha }=e_{\;a}^{\alpha }E_{\beta }^{\;b}X_{b}^{a}$$

as transformations between the coordinate frame and the vielbein frame.

The mapping of $M\times R\;⟶\;M_{5}$ in (21) provides a natural foliation of the tangent space into spacetime hyperspaces of equal τ. Thus, we further partition the quintrad indices, as for the coordinate indices, so that the index convention becomes

(64) $$\begin{array}{ccc} \alpha ,\beta ,\gamma ,\delta =0,1,2,3,5 & \; & \lambda ,\mu ,\nu ,\rho ,\ldots =0,1,2,3\\ a,b,c,d=0,1,2,3,5 & \; & k,l,m,n,\ldots =0,1,2,3 \end{array}$$

where the five index with respect to the quintrad frame will be denoted $\bar{5}$ when necessary to avoid confusion. In this notation the frame transformations can be expanded as

(65) $$\begin{array}{ccc} g_{\mu }=E_{\mu }^{\;k}e_{k}+E_{\mu }^{\;\bar{5}}e_{5}\; & \; & e_{k}=e_{\;k}^{\mu }g_{\mu }+e_{\;k}^{5}g_{5}\\ g_{5}=E_{5}^{\;k}e_{k}+E_{5}^{\;\bar{5}}e_{5}\; & \; & e_{5}=e_{\;\bar{5}}^{\mu }g_{\mu }+e_{\;\bar{5}}^{5}g_{5} \end{array}$$

and

(66) $$\begin{array}{ccc} g^{\mu }=e_{\;k}^{\mu }e^{k}+e_{\;\bar{5}}^{\mu }e^{5}\; & \; & e^{k}=E_{\mu }^{\;k}g^{\mu }+E_{5}^{\;k}g^{5}\\ g^{5}=e_{\;k}^{5}e^{k}+e_{\;\bar{5}}^{5}e^{5}\; & \; & e^{5}=E_{\mu }^{\;\bar{5}}g^{\mu }+E_{5}^{\;\bar{5}}g^{5} \end{array}$$

where the orthogonality relations (58) are required for consistency. In the quintrad we expect the spacetime hypersurface to be spanned by $\left\{e_{k}\right\}$ and normal to $e_{5}$, which points in the direction of advancing τ. We therefore denote the unit normal to spacetime and its associated 1-form as

(67) $$n=e_{5}\;\;\;\bar{n}=\sigma \;e^{5}$$

with normalizations

(68) $$n^{2}=e_{5}\cdot e_{5}=\sigma \;\;{\bar{n}}^{2}=e^{5}\cdot e^{5}=\sigma .$$

Introducing the ADM parameterization [19] we express the fifth basis vector for $T\left(M_{5}\right)$ as a linear combination of *n* and $\left\{g_{\mu }\right\}$

(69) $$g_{5}=N^{\mu }g_{\mu }+Nn$$

where the 4-vector $N^{\mu }$ generalizes the shift 3-vector in 3+1 formalisms and *N* is the lapse function with respect to τ. Designating the spacetime part of the 5D coordinate frame metric as

(70) $$\gamma_{\mu \nu }=g_{\mu \nu }=g_{\mu }\cdot g_{\nu }$$

we can express the 5-components of the coordinate metric as

(71) $$\begin{array}{c} g_{5\mu }=g_{\mu }\cdot g_{5}=\gamma_{\mu \mu^{\prime }}N^{\mu^{\prime }}=N_{\mu }\\ g_{55}=\left(N^{\mu }g_{\mu }+Nn\right)\cdot \left(N^{\mu^{\prime }}g_{\mu^{\prime }}+Nn\right)=\gamma_{\mu \mu^{\prime }}N^{\mu }N^{\mu^{\prime }}+\sigma N^{2} \end{array}$$

so that

(72) $$g_{\alpha \beta }=\left[\begin{array}{cc} \gamma_{\mu \nu } & N_{\mu }\\ N_{\mu } & \sigma N^{2}+\gamma_{\mu \nu }N^{\mu }N^{\nu } \end{array}\right]$$

with

(73) $$g^{\alpha \beta }=\left[\begin{array}{cc} \gamma^{\mu \nu }+\sigma \frac{1}{N^{2}}N^{\mu }N^{\nu } & -\sigma \frac{1}{N^{2}}N^{\mu }\\ -\sigma \frac{1}{N^{2}}N^{\mu } & \sigma \frac{1}{N^{2}} \end{array}\right]$$

as its inverse.

With these choices we have

(74) $$e_{5}=n=\frac{1}{N}\left(-N^{\mu }g_{\mu }+g_{5}\right)\;\;e^{5}=\sigma \bar{n}=\sigma^{2}N\;g^{5}=N\;g^{5}$$

so that comparison with (65) and (66) determines four vielbein components

(75) $$e_{\;\bar{5}}^{\mu }=-\frac{1}{N}N^{\mu }\;\;e_{\;\bar{5}}^{5}=\frac{1}{N}\;\;E_{\mu }^{\;\bar{5}}=0\;\;E_{5}^{\;\bar{5}}=N.$$

Orthogonality in the quintrad frame provides two additional conditions

(76) $$\begin{array}{cc} 0 & =e_{k}\cdot e_{5}=\left(e_{\;k}^{\mu }g_{\mu }+e_{\;k}^{5}g_{5}\right)\cdot \frac{1}{N}\left(-N^{\mu^{\prime }}g_{\mu^{\prime }}+g_{5}\right)\\ & =\frac{1}{N}\left(-e_{\;k}^{\mu }N_{\mu }+e_{\;k}^{\mu }g_{\mu 5}-e_{\;k}^{5}g_{\mu^{\prime }5}N^{\mu^{\prime }}+e_{\;k}^{5}g_{55}\right) \end{array}$$

and

(77) $$0=e^{k}\cdot e^{5}=\left(E_{\mu }^{\;k}g^{\mu }+E_{5}^{\;k}g^{5}\right)\cdot N\;g^{5}=N\left(E_{\mu }^{\;k}g^{\mu 5}+E_{5}^{\;k}g^{55}\right),$$

which combined with (71) provide the components

(78) $$e_{\;k}^{5}=0\;\;E_{5}^{\;k}=E_{\mu }^{\;k}N^{\mu }.$$

Inserting $e_{\;k}^{5}=0$ into (65) leads to

(79) $$e_{k}=e_{\;k}^{\mu }g_{\mu }+e_{\;k}^{5}g_{5}=e_{\;k}^{\mu }\left(E_{\mu }^{\;k^{\prime }}e_{k^{\prime }}+E_{\mu }^{\;\bar{5}}e_{5}\right)$$

from which we conclude that

(80) $$E_{\mu }^{\;\bar{5}}=0\;\;e_{\;k}^{\mu }E_{\mu }^{\;k^{\prime }}=\delta_{k}^{k^{\prime }}.$$

The transformations between coordinate and quintrad frames take the final form

(81) $$\begin{array}{c} g_{\alpha }=E_{\alpha }^{\;a}e_{a}=\delta_{\alpha }^{\mu }E_{\mu }^{\;k}e_{k}+\delta_{\alpha }^{5}\left(E_{\mu }^{\;k}N^{\mu }e_{k}+Ne_{5}\right)\\ e_{a}=e_{\;a}^{\alpha }g_{\alpha }=\delta_{a}^{k}e_{\;k}^{\mu }g_{\mu }+\delta_{a}^{5}\frac{1}{N}\left(-N^{\mu }g_{\mu }+g_{5}\right) \end{array}$$

providing a quintrad basis for $T\left(M_{5}\right)$ with a 4+1 foliation built in by construction. The vielbein field may be summarized as

(82) $$\begin{array}{c} E_{\alpha }^{\;a}=\delta_{\alpha }^{\mu }\delta_{k}^{a}E_{\mu }^{\;k}+\delta_{\alpha }^{5}\left(E_{\mu }^{\;k}N^{\mu }\delta_{k}^{a}+N\delta_{5}^{a}\right)\\ e_{\;a}^{\alpha }=\delta_{a}^{k}\delta_{\mu }^{\alpha }e_{\;k}^{\mu }-\delta_{a}^{5}\delta_{\mu }^{\alpha }\frac{1}{N}N^{\mu }+\delta_{a}^{5}\delta_{5}^{\alpha }\frac{1}{N} \end{array}$$

and since $e_{\;k}^{\mu }=E_{\;k}^{\mu }$ by orthogonality, the vielbein is constructed from $E_{\;k}^{\mu }$, *N* and $N^{\mu }$. As shown in Section 4 and Section 5, the τ-dependent lapse *N* and shift $N^{\mu }$ play the role of Lagrange multipliers, enforcing the constraints on $g_{\alpha \beta }$ associated with the Bianchi relations, but they are not subject to second-order evolution equations. Thus, the dynamical content of the vielbein field is entirely contained in the spacetime part $E_{\mu }^{\;k}$, where μ,k=0,1,2,3.

### 3.2. Field Equations

The standard Einstein field equations

(83) $$R_{\mu \nu }-\frac{1}{2}g_{\mu \nu }R=\frac{8\pi G}{c^{4}}T_{\mu \nu }$$

enjoy O(3,1) covariance, general diffeomorphism invariance, and the Bianchi identity for the Ricci tensor. It follows from the Bianchi identity that of the ten components of (83) four represent constraints [31]. This is made explicit the in 3+1 ADM formalism, which decomposes the field equations into a pair of coupled *t*-evolution equations for the geometry of 3D space, along with 3-vector and scalar constraints among the initial conditions. As articulated by Wheeler [33], “A decade and more of work by Dirac, Bergmann, Schild, Pirani, Anderson, Higgs, Arnowitt, Deser, Misner, DeWitt, and others has taught us through many a hard knock that Einstein’s geometrodynamics deals with the dynamics of geometry: of 3-geometry, not 4-geometry.”

The 3+1 ADM decomposition is most conveniently performed using the trace-reversed form of (83) found from

(84) $$g^{\mu \nu }R_{\mu \nu }-\frac{1}{2}g^{\mu \nu }g_{\mu \nu }R=\frac{8\pi G}{c^{4}}g^{\mu \nu }T_{\mu \nu }$$

and using $g^{\mu \nu }g_{\mu \nu }=4$ to obtain

(85) $$R_{\mu \nu }=\frac{8\pi G}{c^{4}}\left(T_{\mu \nu }-\frac{1}{2}g_{\mu \nu }T\right)$$

which expresses the relationship between spacetime geometry on the LHS and the distribution of matter on the RHS. But in 5D we have $g^{\alpha \beta }g_{\alpha \beta }=5\neq 4$, leading to the trace-reversed form

(86) $$R_{\alpha \beta }=\frac{8\pi G}{c^{4}}\left(T_{\alpha \beta }+\frac{\frac{1}{2}g_{\alpha \beta }}{1-\frac{1}{2}g^{\gamma \delta }g_{\gamma \delta }}g^{\gamma^{\prime }\delta^{\prime }}T_{\gamma^{\prime }\delta^{\prime }}\right)=\frac{8\pi G}{c^{4}}\left(T_{\alpha \beta }-\frac{1}{3}g_{\alpha \beta }T\right).$$

As we shall see in Section 6, this form cannot be made consistent with standard phenomenology in weak SHP gravitation, where the metric may be found as an additive perturbation $h_{\alpha \beta }$ to the flat space metric $\eta_{\alpha \beta }$. Correct perturbative solutions require the replacement $\;\eta_{\alpha \beta }⟶{\hat{\eta }}_{\alpha \beta }=\left(-1,1,1,1,0\right)\;$ in the trace-reversed matter terms, which breaks the apparent 5D symmetry of the source to O(3,1). For the full field equations we expect a similar substitution $g_{\alpha \beta }⟶{\hat{g}}_{\alpha \beta }$ on the RHS of (86), where the modified metric remains to be determined. By requiring ${\hat{g}}^{\;\alpha \beta }\;\;{\hat{g}}_{\;\alpha \beta }=4$ we recover the form

(87) $$R_{\alpha \beta }=\frac{8\pi G}{c^{4}}\left(T_{\alpha \beta }-\frac{1}{2}{\hat{g}}_{\;\alpha \beta }\hat{T}\right)$$

where $\hat{T}={\hat{g}}^{\alpha \beta }T_{\alpha \beta }$.

To find an expression for ${\hat{g}}_{\alpha \beta }$ we write unbroken 5D Einstein equations in the quintrad frame

(88) $$R_{ab}-\frac{1}{2}\eta_{ab}R=\frac{8\pi G}{c^{4}}T_{ab}\;⟶\;R_{ab}=\frac{8\pi G}{c^{4}}\left(T_{ab}+\frac{\frac{1}{2}\eta_{ab}}{1-\frac{1}{2}\eta^{cd}\eta_{cd}}\eta^{c^{\prime }d^{\prime }}T_{c^{\prime }d^{\prime }}\right)$$

where the metric $\eta_{ab}$ is flat, while $R_{ab}$ and $T_{ab}$ are related to the coordinate frame tensors through the vielbein field. We break the 5D symmetry in the matter terms by replacing

(89) $$\eta_{ab}⟶{\hat{\eta }}_{ab}=\left(-1,1,1,1,0\right)=\delta_{a}^{k}\delta_{b}^{l}\;\;\eta_{kl}$$

on the RHS of (88), leaving the Ricci tensor $R_{ab}$ unchanged. The gravitational Einstein equations in the SHP formalism now take the form

(90) $$R_{ab}=\frac{8\pi G}{c^{4}}\left(T_{ab}-\frac{1}{2}{\hat{\eta }}_{ab}\hat{T}\right)$$

where $\hat{T}={\hat{\eta }}^{\;ab}T_{ab}=\eta^{kl}T_{kl}$. Using the vielbein field (82) we may transform (90) back to a coordinate frame, leading to

(91) $$R_{\alpha \beta }=\frac{8\pi G}{c^{4}}\left(T_{\alpha \beta }-\frac{1}{2}{\hat{g}}_{\alpha \beta }\hat{T}\right)$$

providing the symmetry-broken local metric as

(92) $${\hat{g}}_{\alpha \beta }=E_{\alpha }^{\;a}E_{\beta }^{\;b}\;{\hat{\eta }}_{ab}=g_{\alpha \beta }-\delta_{\alpha }^{5}\delta_{\beta }^{5}\eta_{55}N^{2}=g_{\alpha \beta }-\sigma n_{\alpha }n_{\beta }=P_{\alpha \beta }.$$

We notice that $P_{\alpha \beta }$ is a projection operator onto the spacetime hypersurface in $T\left(M_{5}\right)$, and satsifies

(93) $$P_{\alpha \beta }n^{\beta }=0\;\;P_{\alpha \beta }P^{\beta \gamma }=P_{\alpha \beta }\left(g^{\beta \gamma }-\sigma n^{\beta }n^{\gamma }\right)=g_{\alpha }^{\gamma }-\sigma n_{\alpha }n^{\gamma }=P_{\alpha }^{\gamma }$$

from which we may write the completeness relation

(94) $$\delta_{\alpha }^{\beta }=g_{\alpha }^{\beta }=P_{\alpha }^{\beta }+\sigma n_{\alpha }n^{\beta }.$$

The breaking of 5D symmetry can thus be understood as replacing the metric in the matter terms of the field equation with the projector onto the 4D hypersurface.

Using the completeness relation (94), we may decompose the mass–energy–momentum tensor into

(95) $$T_{\alpha \beta }=T_{\alpha^{\prime }\beta^{\prime }}\left(P_{\alpha }^{\alpha^{\prime }}+\sigma n^{\alpha \alpha \prime }n_{\alpha }\right)\left(P_{\beta }^{\beta^{\prime }}+\sigma n^{\beta^{\prime }}n_{\beta }\right)=S_{\alpha \beta }-2\sigma n_{\alpha }p_{\beta }+n_{\alpha }n_{\beta }\kappa$$

where

(96) $$S_{\alpha \beta }=P_{\alpha }^{\alpha^{\prime }}P_{\beta }^{\beta^{\prime }}T_{\alpha^{\prime }\beta^{\prime }}\;\;p_{\beta }=-n^{\alpha }P_{\beta }^{\beta^{\prime }}T_{\alpha \beta^{\prime }}\;\;\kappa =n^{\beta }n^{\alpha }T_{\alpha \beta }$$

representing the 4D energy–momentum tensor $S_{\alpha \beta }$ found by projecting $T_{\alpha \beta }$ onto the spacetime hypersurface, a 4-momentum vector $p_{\beta }$ describing the flow of mass into spacetime, and an invariant form κ of the scalar mass density $T_{55}$. The trace of $T_{\alpha \beta }$ is

(97) $$T=\eta^{\alpha \beta }T_{\alpha \beta }=S+\sigma \kappa$$

but the symmetry-broken trace is

(98) $$\hat{T}={\hat{g}}^{\alpha \beta }T_{\alpha \beta }=P^{\alpha \beta }T_{\alpha \beta }=P^{\alpha \beta }\left(S_{\alpha \beta }+2\sigma n_{\alpha }p_{\beta }+n_{\alpha }n_{\beta }\kappa \right)=S$$

where we used $P^{\alpha \beta }n_{\alpha }=0$. The SHP field equations in a coordinate frame now take the form

(99) $$R_{\alpha \beta }=\frac{8\pi G}{c^{4}}\left(T_{\alpha \beta }-\frac{1}{2}P_{\alpha \beta }S\right).$$

## 4. Decomposition, Projection, and Initial Value Problem

As we will see in this section, the fifteen degrees of freedom in the symmetric 5D Einstein tensor

(100) $$G_{\alpha \beta }=R_{\alpha \beta }-\frac{1}{2}g_{\alpha \beta }R$$

can be decomposed into ten components $G_{\mu \nu }$ satisfying second-order differential equations and five components $G_{5\mu }$ that impose τ-dependent constraints satisfying algebraic equations. The differential equations require as initial conditions $\gamma_{\mu \nu }\left(x,\tau_{0}\right)$ and $\partial_{\tau }\gamma_{\mu \nu }\left(x,\tau_{0}\right)$ at some $\tau_{0}$.

A plausible argument for this decomposition can be seen [34] by writing the Bianchi relations as

(101) $$\nabla_{\alpha }G^{\alpha \beta }=\partial_{\alpha }G^{\alpha \beta }+\mathrm{Christoffel}\;\mathrm{Symbols}\times G^{\alpha \beta }=0$$

forming a set of relations among the field entities. Expanding $\partial_{\alpha }G^{\alpha \beta }=\partial_{\mu }G^{\mu \beta }+\partial_{5}G^{5\beta }$ to rewrite the Bianchi relations as

(102) $$\frac{1}{c_{5}}\partial_{\tau }G^{5\beta }=-\partial_{\mu }G^{\mu \beta }-\;\mathrm{Christoffel}\;\mathrm{Symbols}\times G^{\alpha \beta }$$

we see that since the LHS cannot be more than second-order in $\partial_{\tau }$, we must conclude that $G^{5\beta }$ cannot be more than first-order in $\partial_{\tau }$. Now, if $G^{\alpha \beta }\left(x,\tau_{0}\right)=0$ then $\partial_{\mu }G^{\alpha \beta }\left(x,\tau_{0}\right)=0$, which leads to

(103) $$\frac{1}{c_{5}}\partial_{\tau }G^{5\beta }\left(x,\tau_{0}\right)=-\partial_{\mu }G^{\mu \beta }\left(x,\tau_{0}\right)-\;\mathrm{Christoffel}\;\mathrm{Symbols}\times G^{\alpha \beta }\left(x,\tau_{0}\right)=0$$

and so any terms contained in $G^{5\beta }$ must be constraints on the initial conditions that propagate to future times with τ, but whose form does not evolve.

The formal decomposition of the field equations is accomplished by way of projection of the relevant geometric structures defined on $M_{5}$ onto M(τ). Among these are intrinsic curvature, which appears in the parallel transport of a vector tangent to the surface, and extrinsic curvature, which appears in the parallel transport of a vector normal to the surface. As an intermediate step we define the scalar field S(X)=τ whose level surfaces provide the natural foliation of pseudo-spacetime

(104) $$\Sigma \left(\tau_{0}\right)\subset M_{5}=\left\{X\in M_{5}\;|\;S\left(X\right)=X^{5}/c_{5}=\tau_{0}\right\}$$

implied by the construction of $M_{5}$ in (21). Since $\Sigma (\tau_{0})$ is homeomorphic to $M(\tau_{0})$ for any $\tau_{0}$, we may drop reference to $\tau_{0}$. Thus, for any vector $V=V^{\alpha }g_{\alpha }\in T\left(M_{5}\right)$, where $g_{\alpha }=\partial_{\alpha }$ is the coordinate basis given in (51), the projection operator (92) provides the vector $V_{⊥}^{\alpha }=P_{\beta }^{\alpha }V^{\beta }\in T\left(\Sigma \right)$. The homeomorphism is expressed by way of the vector $v\in T\left(M\right)$ for which

(105) $$V_{⊥}^{\alpha }=v^{\mu }{\left(g_{\mu }\right)}^{\alpha }=v^{\mu }\;\frac{\partial X^{\alpha }}{\partial x^{\mu }}=v^{\mu }\;\delta_{\mu }^{\alpha }$$

and so

(106) $$V_{⊥}^{5}=0\;⟶\;v^{\mu }=\delta_{\alpha }^{\mu }V_{⊥}^{\alpha }=\delta_{\alpha }^{\mu }P_{\beta }^{\alpha }V^{\beta }=P_{\beta }^{\mu }V^{\beta }.$$

To express the projection operator (92) we transform the unit normal $n=e_{5}$ defined in (67) for the vielbein frame to a coordinate frame as

(107) $$n_{\alpha }=\left[\delta_{\alpha }^{\mu }\delta_{k}^{a}E_{\mu }^{\;k}+\delta_{\alpha }^{5}\left(E_{\mu }^{\;k}N^{\mu }\delta_{k}^{a}+N\delta_{5}^{a}\right)\right]\eta_{a5}=\sigma \;N\;\delta_{\alpha }^{5}$$

where we used $n_{\alpha }=n\cdot e_{\alpha }=e_{5}\cdot e_{\alpha }=\eta_{\alpha 5}$. Similarly,

(108) $$n^{\alpha }=g^{\alpha \beta }n_{\beta }=g^{\alpha \beta }\eta_{\beta 5}=\frac{1}{N}{\left(-N^{\mu }g_{\mu }+g_{5}\right)}^{\alpha }=\frac{1}{N}\left(-N^{\mu }\delta_{\mu }^{\alpha }+\delta_{5}^{\alpha }\right).$$

The extrinsic curvature $K_{\mu \nu }$ will be defined as the projection of the covariant derivative of the unit normal onto M(τ). We will see that $K_{\mu \nu }$ is closely related to $\partial_{\tau }\gamma_{\mu \nu }$.

In the Legendre transformation from Lagrangian to Hamiltonian mechanics, the canonical momentum is introduced as an auxiliary variable, so that the phase space $\left(x,\dot{x}\right)$ becomes (x,p) containing no explicit τ-derivatives. This transforms the equations of motion from one expression involving the second-order derivatives ${\ddot{x}}^{\mu }$ to a pair of expressions involving only the first-order derivatives ${\dot{x}}^{\mu }$ and ${\dot{p}}^{\mu }$. By analogy, the ADM approach to geometrodynamics introduces the extrinsic curvature $K_{\mu \nu }$ as an auxiliary variable, transforming the second-order field Equation (99) in $g_{\alpha \beta }$ into a pair of first-order differential equations for $\gamma_{\mu \nu }$ and $K_{\mu \nu }$ in spacetime. Using standard methods from the theory of embedded surfaces, the initial value problem is posed in two stages. First, we study the evolution of the spacetime hypersurface through the Lie derivative in the direction of the unit normal to Σ, leading to an expression relating $\partial_{\tau }\gamma_{\mu \nu }$ to $K_{\mu \nu }$. We then decompose the Ricci tensor $R_{\alpha \beta }$ into components tangent and normal to Σ, providing an expression relating $\partial_{\tau }K_{\mu \nu }$ to the matter terms in the field equations and a pair of constraints on the initial conditions.

### 4.1. Projected Covariant Derivative and Curvature

With the compatible connection $\Gamma_{\alpha \beta }^{\gamma }$ the covariant derivative of $g_{\alpha \beta }$ on $M_{5}$ vanishes, leading to the Ricci identity

(109) $$\left[\nabla_{\beta },\nabla_{\alpha }\right]X_{\delta }=X_{\gamma }R_{\delta \alpha \beta }^{\gamma }$$

with Riemann tensor

(110) $$R_{\delta \alpha \beta }^{\gamma }=\frac{\partial }{\partial x^{\alpha }}\Gamma_{\delta \beta }^{\gamma }-\frac{\partial }{\partial x^{\beta }}\Gamma_{\delta \alpha }^{\gamma }+\Gamma_{\sigma \alpha }^{\gamma }\Gamma_{\delta \beta }^{\sigma }-\Gamma_{\sigma \beta }^{\gamma }\Gamma_{\delta \alpha }^{\sigma }$$

and associated Bianchi relations. The projected covariant derivative is defined as

(111) $${\bar{\nabla }}_{\alpha }V_{\beta }=P_{\alpha }^{\alpha^{\prime }}P_{\beta }^{\beta^{\prime }}\nabla_{\alpha^{\prime }}V_{\beta^{\prime }}$$

and since

(112) $$\nabla_{\alpha }P_{\beta \gamma }=\nabla_{\alpha }\left(g_{\beta \gamma }-\sigma n_{\beta }n_{\gamma }\right)=-\sigma \nabla_{\alpha }\left(n_{\beta }n_{\gamma }\right)=-\sigma \left[\left(\nabla_{\alpha }n_{\beta }\right)n_{\gamma }+n_{\beta }\nabla_{\alpha }n_{\gamma }\right]$$

we have the compatibility condition

(113) $${\bar{\nabla }}_{\alpha }P_{\beta \gamma }=-\sigma P_{\alpha }^{\alpha^{\prime }}P_{\beta }^{\beta^{\prime }}P_{\gamma }^{\gamma^{\prime }}\left[\left(\nabla_{\alpha^{\prime }}n_{\beta^{\prime }}\right)n_{\gamma^{\prime }}+n_{\beta^{\prime }}\nabla_{\alpha^{\prime }}n_{\gamma^{\prime }}\right]=0$$

where we used $P_{\beta }^{\delta }n_{\delta }=0$. We notice that for a vector $V=V^{⊥}\in T\left(\Sigma \right)$

(114) $${\bar{\nabla }}_{\alpha }V_{\beta }^{⊥}={\bar{\nabla }}_{\alpha }\left(P_{\beta }^{\beta^{\prime }}V_{\beta^{\prime }}\right)=P_{\beta }^{\beta^{\prime }}\;{\bar{\nabla }}_{\alpha }\left(V_{\beta^{\prime }}\right)=P_{\beta }^{\beta^{\prime }}\left(P_{\alpha }^{\alpha^{\prime }}\nabla_{\alpha^{\prime }}\right)V_{\beta^{\prime }}={\bar{\nabla }}_{\alpha }V_{\beta }$$

by comparison with (111). This compatibility justifies regarding ${\bar{\nabla }}_{\alpha }$ as the intrinsic covariant derivative on $T\left(\Sigma \right)$, denoted as

(115) $$D_{\alpha }={\bar{\nabla }}_{\alpha }=P_{\alpha }^{\gamma }\nabla_{\gamma }\;\;D_{\mu }=P_{\mu }^{\alpha }D_{\alpha }=P_{\mu }^{\alpha }P_{\alpha }^{\gamma }\nabla_{\gamma }=P_{\mu }^{\gamma }\nabla_{\gamma }$$

where we used (106), leading to $D_{\mu }\gamma_{\lambda \rho }=0$. The projected curvature ${\bar{R}}_{\lambda \mu \nu }^{\rho }$ is defined through the projected covariant derivatives

(116) $$\left[D_{\nu },D_{\mu }\right]X_{\lambda }=X_{\rho }{\bar{R}}_{\lambda \mu \nu }^{\rho }$$

and will be given an explicit form below.

For vectors $V,U\in T\left(\Sigma \right)\subset T\left(M\right)$, the Weingarten map is defined as

(117) $$\chi \left(V\right)=\nabla_{V}n=V\cdot \left(\nabla n\right)\;\;\chi^{\alpha }\left(V\right)=V^{\beta }\nabla_{\beta }n^{\alpha }$$

and the extrinsic curvature on $T\left(\Sigma \right)$ is defined as the projection onto a vector *U* of the Weingarten map along a vector *V*,

(118) $$\begin{array}{ccc} K\left(U,V\right)= & & \;-U\cdot \chi \left(V\right)=-U\cdot \nabla_{V}n=-g_{\alpha \gamma }V^{\alpha }U^{\beta }\nabla_{\beta }n^{\gamma } \end{array}$$

(119) $$\begin{array}{ccc} K_{\alpha \beta }= & & \;-g_{\alpha \gamma }\nabla_{\beta }n^{\gamma }=-\nabla_{\beta }n_{\alpha }. \end{array}$$

This definition is extended to T(M) as

(120) $$\begin{array}{ccc} K\left(U^{⊥},V^{⊥}\right)= & & \;K\left(PU,PV\right)=-g_{\gamma^{\prime }\gamma }\left(P_{\alpha }^{\gamma^{\prime }}V^{\alpha }\right)\left(P_{\beta^{\prime }}^{\beta }U^{\beta^{\prime }}\right)\nabla_{\beta }n^{\gamma } \end{array}$$

(121) $$\begin{array}{ccc} K_{\beta^{\prime }\alpha }U^{\beta^{\prime }}V^{\alpha }= & & \;V^{\alpha }U^{\beta^{\prime }}\left(-g_{\gamma^{\prime }\gamma }P_{\alpha }^{\gamma^{\prime }}P_{\beta^{\prime }}^{\beta }\right)\nabla_{\beta }n^{\gamma } \end{array}$$

(122) $$\begin{array}{ccc} K_{\alpha \beta }= & & \;-P_{\alpha }^{\alpha^{\prime }}P_{\beta }^{\beta^{\prime }}\;\nabla_{\beta^{\prime }}n_{\alpha^{\prime }} \end{array}$$

where although $n_{\gamma }$ is normal to $T\left(\Sigma \right)$, the covariant derivative $\nabla_{\delta }n_{\gamma }$ may have both normal and tangent components. Using $n^{2}=\sigma$ we have

(123) $$0=\nabla_{\alpha }n^{2}=2n^{\beta }\left(\nabla_{\alpha }n_{\beta }\right)$$

so that

(124) $$P_{\alpha }^{\alpha^{\prime }}\;\nabla_{\beta }n_{\alpha^{\prime }}=\left(g_{\alpha }^{\alpha^{\prime }}-\sigma n_{\alpha }n^{\alpha^{\prime }}\right)\nabla_{\beta }n_{\alpha^{\prime }}=\nabla_{\beta }n_{\alpha }$$

and so (122) becomes

(125) $$K_{\alpha \beta }=-P_{\alpha }^{\alpha^{\prime }}\nabla_{\alpha^{\prime }}n_{\beta }=-\nabla_{\alpha }n_{\beta }+\sigma n_{\alpha }\left(n^{\alpha^{\prime }}\nabla_{\alpha^{\prime }}n_{\beta }\right)$$

with the contracted form

(126) $$K=\gamma^{\alpha \beta }K_{\alpha \beta }=\gamma^{\alpha \beta }P_{\alpha }^{\alpha^{\prime }}P_{\beta }^{\beta^{\prime }}\nabla_{\alpha^{\prime }}n_{\beta^{\prime }}=\gamma^{\alpha \beta }\nabla_{\alpha }n_{\beta }=\nabla_{\alpha }n^{\alpha }.$$

Using (107) for the unit normal $n_{\alpha }$ we expand

(127) $$\begin{array}{ccc} \left(n^{\gamma }\nabla_{\gamma }n_{\beta }\right)= & & \;\sigma n^{\gamma }\nabla_{\gamma }\left(N\nabla_{\beta }\tau \right)=\sigma n^{\gamma }\left(\nabla_{\gamma }N\right)\frac{n_{\beta }}{\sigma N}+\sigma n^{\gamma }N\nabla_{\beta }\left(\frac{n_{\gamma }}{\sigma N}\right)\\ = & & \;\frac{1}{N}\left[n^{\gamma }n_{\beta }\nabla_{\gamma }N-\sigma \delta_{\beta }^{\gamma }\nabla_{\gamma }N\right]=-\sigma \frac{1}{N}\left[\delta_{\beta }^{\gamma }-\sigma n^{\gamma }n_{\beta }\right]\nabla_{\gamma }N\\ = & & \;-\sigma \frac{1}{N}P_{\beta }^{\gamma }\nabla_{\gamma }N=-\sigma \frac{1}{N}D_{\beta }N \end{array}$$

to obtain the extrinsic curvature (125) in the useful form

(128) $$K_{\alpha \beta }=-\nabla_{\alpha }n_{\beta }-n_{\alpha }\frac{1}{N}D_{\beta }N.$$

### 4.2. Evolution of the Hypersurface Σ

With the parameterization (69) the fifth basis vector is

(129) $$\partial_{5}=N^{\mu }g_{\mu }+Nn=N+Nn$$

and we define the normal evolution vector m=Nn, noting that for the time function $S\left(X\right)$, which defines the foliation (104), we have

(130) $$dS\left(X\right)\cdot m=\delta_{\alpha }^{5}{\left(Nn\right)}^{\alpha }=\delta_{\alpha }^{5}N\left(g^{\alpha \beta }\sigma N\;\delta_{\beta }^{5}\right)=N^{2}\sigma g^{55}=N^{2}\sigma \left(\sigma \frac{1}{N^{2}}\right)=1.$$

Under a displacement

(131) $$X⟶X^{\prime }=X+m\;\delta \tau \left(X\right)$$

the time function evolves as

(132) $$S\left(X\right)⟶S\left(X^{\prime }\right)=S\left(X+m\;\delta \tau \right)=S\left(X\right)+dS\left(X\right)\cdot m\;\delta \tau =S\left(X\right)+\delta \tau$$

showing that for $X\in \Sigma_{\tau }$

(133) $$X^{\prime }=X+m\;\delta S\left(X\right)\in \Sigma_{\tau +\delta \tau }$$

so that the hypersurface $\Sigma_{\tau +\delta \tau }$ is obtained from $\Sigma_{\tau }$ by the displacement of each point by mδS. The evolution of the hypersurface Σ can thus be characterized through the Lie derivative in the direction of the normal evolution vector

(134) $$L_{m}=L_{5}-L_{N}.$$

The Lie derivative of a second rank tensor $A_{\alpha \beta }$ is

(135) $$L_{m}A_{\alpha \beta }=m^{\gamma }\nabla_{\gamma }A_{\alpha \beta }+A_{\gamma \beta }\nabla_{\alpha }m^{\gamma }+A_{\alpha \gamma }\nabla_{\beta }m^{\gamma }$$

where in the absence of torsion $\nabla_{\gamma }$ can be replaced by $\partial_{\gamma }$ and we have

(136) $$L_{m}\;\delta_{\beta }^{\alpha }=-\nabla_{\beta }m^{\alpha }+\nabla_{\beta }m^{\alpha }=0\;\;L_{m}g_{\alpha \beta }=\nabla_{\alpha }m_{\beta }+\nabla_{\beta }m_{\alpha }$$

(137) $$L_{5}A_{\alpha \beta }=\delta_{5}^{\gamma }\partial_{\gamma }A_{\alpha \beta }+A_{\gamma \beta }\partial_{\alpha }\delta_{5}^{\gamma }+A_{\alpha \gamma }\partial_{\beta }\delta_{5}^{\gamma }=\partial_{5}A_{\alpha \beta }=\frac{1}{c_{5}}\partial_{\tau }A_{\alpha \beta }.$$

For $X\in \Sigma \subset M_{5}$, the squared interval is

(138) $${\left.\delta X^{2}\right|}_{\Sigma }={\left.g_{\alpha \beta }\delta X^{\alpha }\delta X^{\beta }\right|}_{\Sigma }=g_{\alpha \beta }\frac{\partial X^{\alpha }}{\partial x^{\mu }}\frac{\partial X^{\beta }}{\partial x^{\nu }}\delta x^{\mu }\delta x^{\nu }=g_{\alpha \beta }\delta_{\mu }^{\alpha }\delta_{\nu }^{\beta }\delta x^{\mu }\delta x^{\nu }=\gamma_{\mu \nu }\delta x^{\mu }\delta x^{\nu }$$

and since

(139) $$P_{\mu \nu }=g_{\mu \nu }-\sigma n_{\mu }n_{\nu }=\gamma_{\mu \nu }$$

we may find the Lie derivative of the metric $\gamma_{\mu \nu }$ on M by evaluating $L_{m}P_{\alpha \beta }$ and pulling back the result to M. Writing

(140) $$L_{m}\;P_{\alpha \beta }=m^{\gamma }\nabla_{\gamma }P_{\alpha \beta }+P_{\gamma \beta }\nabla_{\alpha }m^{\gamma }+P_{\alpha \gamma }\nabla_{\beta }m^{\gamma }$$

we find

(141) $$m^{\gamma }\nabla_{\gamma }P_{\alpha \beta }=Nn^{\gamma }\nabla_{\gamma }\left(g_{\alpha \beta }-\sigma n_{\alpha }n_{\beta }\right)=-\sigma N\left(n^{\gamma }\left(\nabla_{\gamma }n_{\alpha }\right)n_{\beta }+n_{\alpha }n^{\gamma }\nabla_{\gamma }n_{\beta }\;\right)$$

and using (128) to obtain

(142) $$\nabla_{\beta }m_{\alpha }=N\nabla_{\beta }n_{\alpha }+n_{\alpha }\nabla_{\beta }N=-NK_{\beta \alpha }-n_{\beta }\nabla_{\alpha }N+n_{\alpha }\nabla_{\beta }N$$

we finally arrive at

(143) $$L_{m}\;\gamma_{\mu \nu }=L_{5}\;\gamma_{\mu \nu }-L_{N}\;\gamma_{\mu \nu }$$

from which we can write

(144) $$\frac{1}{c_{5}}\partial_{\tau }\gamma_{\mu \nu }=L_{N}\;\gamma_{\mu \nu }-2NK_{\mu \nu }$$

as the evolution equation for the metric.

### 4.3. Decomposition of the Riemann Tensor

The 4+1 decomposition of $R_{\;\;\delta \alpha \beta }^{\gamma }$ is accomplished by projecting onto Σ and *n*. Using the completeness relation (94) to write

(145) $$R_{\;\;\delta \alpha \beta }^{\gamma }=\left(P_{\alpha }^{\alpha^{\prime }}+\sigma n_{\alpha }n^{\alpha^{\prime }}\right)\left(P_{\beta }^{\beta^{\prime }}+\sigma n_{\beta }n^{\beta^{\prime }}\right)\left(P_{\gamma^{\prime }}^{\gamma }\;+\sigma n^{\gamma }n_{\gamma^{\prime }}\right)\left(P_{\delta }^{\delta^{\prime }}\;+\sigma n_{\delta }n^{\delta^{\prime }}\right)R_{\;\;\delta^{\prime }\alpha^{\prime }\beta^{\prime }}^{\gamma^{\prime }}$$

we obtain products of the type

(146) $$R_{\delta \alpha \beta }^{\gamma }=\delta_{\alpha }^{\alpha^{\prime }}\;\;\delta_{\beta }^{\beta^{\prime }}\;\;\delta_{\gamma^{\prime }}^{\gamma }\;\;\delta_{\delta }^{\delta^{\prime }}\;\;R_{\delta^{\prime }\alpha^{\prime }\beta^{\prime }}^{\gamma^{\prime }}⟶\left\{\begin{array}{c} \delta_{\mu }^{\alpha }\;\;\delta_{\nu }^{\beta }\;\;\delta_{\gamma }^{\lambda }\;\;\delta_{\sigma }^{\delta }\;\;P_{\alpha }^{\alpha^{\prime }}\;\;P_{\beta }^{\beta^{\prime }}\;\;P_{\gamma^{\prime }}^{\gamma }\;\;P_{\delta }^{\delta^{\prime }}\;\;\;\;R_{\delta^{\prime }\alpha^{\prime }\beta^{\prime }\gamma^{\prime }}=R_{\sigma \mu \nu }^{\lambda }\\ \delta_{\mu }^{\alpha }\;\;\delta_{\nu }^{\beta }\;\;\delta_{\gamma }^{\lambda }\;\;P_{\gamma^{\prime }}^{\gamma }n^{\delta }\;\;P_{\alpha }^{\alpha^{\prime }}\;\;P_{\beta }^{\beta^{\prime }}\;\;R_{\delta \alpha^{\prime }\beta^{\prime }}^{\gamma^{\prime }}=\sigma N\;\;R_{5\mu \nu }^{\lambda }\\ \delta^{\alpha \mu }\;\;\delta_{\nu }^{\beta }\;\;P_{\alpha \alpha^{\prime }}\;\;n^{\delta }\;\;P_{\beta }^{\beta^{\prime }}\;\;n^{\gamma }\;\;R_{\delta \beta^{\prime }\gamma }^{\alpha^{\prime }}=N^{2}\;\;R_{5\nu 5}^{\mu } \end{array}\right.$$

where the symmetries of the Riemann tensor lead to $R_{\delta \alpha \beta }^{\gamma }\;\;n^{\delta }\;\;n^{\alpha }\;\;n^{\beta }=0\;$. To expand the projected curvature defined in (116), we write

(147) $$D_{\alpha }D_{\beta }V^{\gamma }=D_{\alpha }\left(D_{\beta }V^{\gamma }\right)=P_{\alpha }^{\alpha^{\prime }}P_{\beta }^{\beta^{\prime }}P_{\gamma }^{\gamma^{\prime }}\nabla_{\alpha^{\prime }}\left(D_{\beta^{\prime }}V^{\gamma^{\prime }}\right)$$

and use (112) to find

(148) $$D_{\alpha }D_{\beta }V^{\gamma }=\sigma K_{\alpha \beta }P_{\gamma^{\prime }}^{\gamma }n^{\beta^{\prime }}\nabla_{\beta^{\prime }}V^{\gamma^{\prime }}+\sigma K_{\alpha }^{\gamma }K_{\beta \delta }V^{\delta }+P_{\alpha }^{\alpha^{\prime }}P_{\beta }^{\beta^{\prime \prime }}P_{\gamma^{\prime \prime }}^{\gamma }\left(\nabla_{\alpha^{\prime }}\nabla_{\beta^{\prime \prime }}V^{\gamma^{\prime \prime }}\right).$$

Thus,

(149) $$\left[D_{\alpha },D_{\beta }\right]V^{\gamma }={\bar{R}}_{\delta \alpha \beta }^{\gamma }V^{\delta }=-\sigma \left(K_{\alpha \delta }K_{\;\;\beta }^{\gamma }-K_{\beta \delta }K_{\;\;\alpha }^{\gamma }\right)V^{\delta }+P_{\;\;\alpha }^{\alpha^{\prime }}P_{\;\;\beta }^{\beta^{\prime }}P_{\;\;\gamma^{\prime }}^{\gamma }\;R_{\;\;\delta^{\prime }\alpha^{\prime }\beta^{\prime }}^{\gamma^{\prime }}P_{\;\;\delta }^{\delta^{\prime }}V^{\delta }$$

which by the quotient theorem on Σ leads to

(150) $$P_{\;\;\alpha }^{\alpha^{\prime }}P_{\;\;\beta }^{\beta^{\prime }}P_{\;\;\gamma^{\prime }}^{\gamma }\;P_{\;\;\delta }^{\delta^{\prime }}R_{\;\;\delta^{\prime }\alpha^{\prime }\beta^{\prime }}^{\gamma^{\prime }}={\bar{R}}_{\delta \alpha \beta }^{\gamma }-\sigma \left(K_{\alpha }^{\gamma }K_{\beta \delta }-K_{\beta }^{\gamma }K_{\alpha \delta }\right)$$

generalizing the Gauss relation. Acting on this expression with $\delta_{\gamma }^{\mu }\delta_{\nu }^{\delta }\delta_{\lambda }^{\alpha }\delta_{\rho }^{\beta }$ we find

(151) $$R_{\;\;\nu \lambda \rho }^{\mu }={\bar{R}}_{\;\;\nu \lambda \rho }^{\mu }-\sigma \left(K_{\lambda }^{\mu }K_{\rho \nu }-K_{\rho }^{\mu }K_{\lambda \nu }\right)$$

providing an expression for the projected curvature ${\bar{R}}_{\;\;\nu \lambda \rho }^{\mu }$ defined in (116) in terms of the spacetime components of the 5D intrinsic curvature $R_{\;\;\nu \lambda \rho }^{\mu }$ and the extrinsic curvature $K_{\rho \nu }$. Contracting on α and γ in (150) leads to

(152) $$P_{\;\;\alpha }^{\alpha^{\prime }}P_{\;\;\beta }^{\beta^{\prime }}R_{\;\;\alpha^{\prime }\beta^{\prime }}-\sigma P_{\alpha \alpha^{\prime }}n^{\delta^{\prime }}P_{\;\;\beta }^{\beta^{\prime }}n^{\gamma \prime }R_{\;\;\delta^{\prime }\beta^{\prime }\gamma^{\prime }}^{\alpha^{\prime }}={\bar{R}}_{\alpha \beta }-\sigma \left(KK_{\alpha \beta }-K_{\alpha }^{\delta }K_{\beta \delta }\right)$$

and contracting on α and β gives

(153) $$R-2\sigma R_{\alpha \beta }n^{\alpha }n^{\beta }=\bar{R}-\sigma \left(K^{2}-K^{\alpha \beta }K_{\alpha \beta }\right)$$

known as the scalar Gauss relation. Some insight into (150) may be found by considering that the curvature ${\bar{R}}_{\;\;\nu \lambda \rho }^{\mu }$ of a hypersurface embedded in a flat space with $R_{\;\;\nu \lambda \rho }^{\mu }=0$ is determined entirely by the extrinsic curvature.

Applying the Ricci identity (109) to the vector *n* as

(154) $$\left(\nabla_{\beta }\nabla_{\alpha }-\nabla_{\alpha }\nabla_{\beta }\right)n^{\gamma }=R_{\gamma^{\prime }\alpha \beta }^{\gamma }n^{\gamma^{\prime }},$$

projecting the LHS onto Σ as

(155) $$P_{\;\;\alpha }^{\alpha^{\prime }}P_{\;\;\beta }^{\beta^{\prime }}P_{\gamma^{\prime }}^{\gamma }\left(\nabla_{\alpha^{\prime }}\nabla_{\beta^{\prime }}-\nabla_{\beta^{\prime }}\nabla_{\alpha^{\prime }}\right)n^{\gamma^{\prime }},$$

and using the identity (125) leads us to

(156) $$D_{\beta }K_{\alpha }^{\gamma }-D_{\alpha }K_{\beta }^{\gamma }=P_{\gamma^{\prime }}^{\gamma }n^{\delta }P_{\;\;\alpha }^{\alpha^{\prime }}P_{\;\;\beta }^{\beta^{\prime }}R_{\delta \alpha^{\prime }\beta^{\prime }}^{\gamma^{\prime }}$$

which is called the Codazzi relation. Using (107) for the unit normal $n_{\alpha }$ provides an interpretation of this expression as

(157) $$n_{\delta }R_{\mu \nu \lambda }^{\delta }=\sigma N\delta_{\delta }^{5}R_{\mu \nu \lambda }^{\delta }⟶R_{\mu \nu \lambda }^{5}=\sigma \frac{1}{N}\left(D_{\lambda }K_{\nu \mu }-D_{\nu }K_{\lambda \mu }\right)$$

recalling the role of the extrinsic curvature $K_{\mu \nu }$ as the curvature of M mapped to the hypersurface Σ and embedded in the larger manifold $M_{5}$. Contracting on α and γ in (156) produces

(158) $$D_{\beta }K-D_{\alpha }K_{\beta }^{\alpha }=n^{\alpha }P_{\;\;\beta }^{\beta^{\prime }}R_{\alpha \beta^{\prime }}$$

known as the contracted Codazzi relation.

Returning to the Ricci identity for $n^{\alpha }$, we apply (128) twice to terms $\nabla_{\beta }\nabla_{\gamma }n^{\alpha }$ and project onto (154) with $P_{\alpha \alpha^{\prime }}n^{\gamma^{\prime }}P_{\beta }^{\beta^{\prime }}$ to obtain

(159) $$-K_{\alpha \gamma }K_{\;\;\beta }^{\gamma }+\sigma \frac{1}{N}D_{\beta }D_{\alpha }N+P_{\;\;\alpha }^{\alpha^{\prime }}P_{\;\;\beta }^{\beta^{\prime }}\;n^{\gamma }\nabla_{\gamma }K_{\alpha^{\prime }\beta^{\prime }}=P_{\alpha \alpha^{\prime }}n^{\gamma^{\prime }}P_{\beta }^{\beta^{\prime }}\;R_{\delta \beta^{\prime }\gamma^{\prime }}^{\alpha^{\prime }}n^{\delta }.$$

Writing the Lie derivative of $K_{\alpha \beta }$ and using (142) for $\nabla_{\beta }m_{\alpha }$

(160) $$L_{m}\;K_{\alpha \beta }=Nn^{\gamma }\nabla_{\gamma }K_{\alpha \beta }-2NK_{\alpha \gamma }K_{\;\;\beta }^{\gamma }-K_{\alpha \gamma }D^{\gamma }Nn_{\beta }-K_{\beta \gamma }D^{\gamma }Nn_{\alpha }$$

the last two equations combine as

(161) $$\frac{1}{N}L_{m}\;K_{\alpha \beta }+\sigma \frac{1}{N}D_{\alpha }D_{\beta }N+K_{\alpha \gamma }K_{\;\;\beta }^{\gamma }=P_{\alpha \alpha^{\prime }}\;n^{\delta }P_{\;\;\beta }^{\beta^{\prime }}\;n^{\gamma }\;\;R_{\;\;\delta \beta^{\prime }\gamma }^{\alpha^{\prime }}$$

to provide an evolution equation for $K_{\alpha \beta }$. Rewriting the contracted Gauss relation (152) as

(162) $$P_{\alpha \alpha^{\prime }}n^{\delta^{\prime }}P_{\;\;\beta }^{\beta^{\prime }}n^{\gamma \prime }R_{\;\;\delta^{\prime }\beta^{\prime }\gamma^{\prime }}^{\alpha^{\prime }}=\sigma P_{\;\;\alpha }^{\alpha^{\prime }}P_{\;\;\beta }^{\beta^{\prime }}R_{\;\;\alpha^{\prime }\beta^{\prime }}-\sigma {\bar{R}}_{\alpha \beta }+KK_{\alpha \beta }-K_{\alpha }^{\delta }K_{\beta \delta }$$

we can put (161) into the form

(163) $$P_{\;\;\alpha }^{\alpha^{\prime }}P_{\;\;\beta }^{\beta^{\prime }}R_{\;\;\alpha^{\prime }\beta^{\prime }}=\frac{1}{N}L_{m}\;K_{\alpha \beta }+\sigma \frac{1}{N}D_{\alpha }D_{\beta }N+{\bar{R}}_{\alpha \beta }-\sigma KK_{\alpha \beta }+\sigma 2K_{\alpha }^{\delta }K_{\beta \delta }$$

in which only $R_{\alpha \beta }$ on the LHS refers explicitly to the 5D geometry of $M_{5}$.

### 4.4. Decomposition of the Field Equations

Moving from the mathematics of embedded surfaces to the physics of gravitation, we introduce the field Equations (99) into (163) by projecting

(164) $$P_{\;\;\alpha }^{\alpha^{\prime }}P_{\;\;\beta }^{\beta^{\prime }}R_{\;\;\alpha^{\prime }\beta^{\prime }}=\frac{8\pi G}{c^{4}}P_{\alpha }^{\alpha^{\prime }}P_{\beta }^{\beta^{\prime }}\left(T_{\alpha^{\prime }\beta^{\prime }}-\frac{1}{2}P_{\alpha^{\prime }\beta^{\prime }}S\right)=\frac{8\pi G}{c^{4}}\left(S_{\alpha \beta }-\frac{1}{2}P_{\alpha \beta }S\right)$$

and pulling back to M to obtain the evolution equation for $K_{\mu \nu }$ in the form

(165) $$\begin{array}{ccc} \frac{1}{c_{5}}\partial_{\tau }K_{\mu \nu }= & & \;L_{N}K_{\mu \nu }-\sigma D_{\mu }D_{\nu }N\\ & & +N\left[-\sigma {\bar{R}}_{\mu \nu }+KK_{\mu \nu }-2K_{\mu }^{\lambda }K_{\nu \lambda }+\sigma \frac{8\pi G}{c^{4}}\left(S_{\mu \nu }-\frac{1}{2}\gamma_{\mu \nu }S\right)\right] \end{array}$$

where again we used $L_{m}=L_{5}-L_{N}$.

The double projection of the field equation onto the unit normal *n* is

(166) $$R_{\alpha \beta }n^{\alpha }n^{\beta }=\frac{8\pi G}{c^{4}}\left(T_{\alpha \beta }-\frac{1}{2}P_{\alpha \beta }S\right)n^{\alpha }n^{\beta }=\frac{8\pi G}{c^{4}}\kappa$$

where we used $P_{\alpha \beta }n^{\alpha }=0$ and the definition of κ in (96). Using the scalar Gauss relation (153)

(167) $$R-2\sigma R_{\alpha \beta }n^{\alpha }n^{\beta }=\bar{R}-\sigma \left(K^{2}-K^{\alpha \beta }K_{\alpha \beta }\right)$$

we evaluate *R* by acting on the field Equation (99) with the symmetry-broken metric $P^{\alpha \beta }$, leading to

(168) $$P^{\alpha \beta }R_{\alpha \beta }=\frac{8\pi G}{c^{4}}P^{\alpha \beta }\left(S_{\alpha \beta }-\frac{1}{2}P_{\alpha \beta }S\right)=-\frac{8\pi G}{c^{4}}S.$$

Finally, the double projection onto *n* of the field is

(169) $$R_{\alpha \beta }n^{\alpha }n^{\beta }=\frac{8\pi G}{c^{4}}\left(T_{\alpha \beta }-\frac{1}{2}P_{\alpha \beta }S\right)n^{\alpha }n^{\beta }=\frac{8\pi G}{c^{4}}T_{\alpha \beta }n^{\alpha }n^{\beta }=\frac{8\pi G}{c^{4}}\kappa$$

so that the scalar Gauss relation becomes

(170) $$\bar{R}-\sigma \left(K^{2}-K^{\mu \nu }K_{\mu \nu }\right)=-\frac{8\pi G}{c^{4}}\left(S+\sigma \kappa \right)$$

known as the Hamiltonian constraint. Since this expression has no τ-derivatives, it will be satisfied at all times if it is satisfied by the initial conditions.

The mixed projection of the field equations with $P_{\;\;\beta }^{\beta^{\prime }}$ and $n^{\alpha }$ is

(171) $$R_{\alpha \beta }n^{\alpha }P_{\;\;\beta }^{\beta^{\prime }}=\frac{8\pi G}{c^{4}}\left(T_{\alpha \beta^{\prime }}-\frac{1}{2}P_{\alpha \beta^{\prime }}S\right)n^{\alpha }P_{\;\;\beta }^{\beta^{\prime }}=\frac{8\pi G}{c^{4}}T_{\alpha \beta^{\prime }}n^{\alpha }P_{\;\;\beta }^{\beta^{\prime }}=-\frac{8\pi G}{c^{4}}p_{\beta }$$

where we used the definition of $p_{\beta }$ in (96). Combining this with the contracted Codazzi relation (158) and pulling back to M leads to the momentum constraint

(172) $$D_{\mu }K_{\nu }^{\mu }-D_{\nu }K=\frac{8\pi G}{c^{4}}p_{\nu }$$

referring to the flow of mass into spacetime. This expression again has no τ-derivatives and remains form-invariant as τ advances. We notice that the evolution equations contain only objects defined on M and so the connection components $\Gamma_{\mu \nu }^{5}$ appear only implicitly through the extrinsic curvature $K_{\mu \nu }$.

Collecting the evolution Equations (144) and (165) with the constraints (170) and (172), the initial value problem takes the final form

$\begin{array}{c} \frac{1}{c_{5}}\partial_{\tau }\gamma_{\mu \nu }=L_{N}\;\gamma_{\mu \nu }-2NK_{\mu \nu }\\ \frac{1}{c_{5}}\partial_{\tau }K_{\mu \nu }=L_{N}K_{\mu \nu }-\sigma D_{\mu }D_{\nu }N\\ \;\;\;+N\left[-\sigma {\bar{R}}_{\mu \nu }+KK_{\mu \nu }-2K_{\mu }^{\lambda }K_{\nu \lambda }+\sigma \frac{8\pi G}{c^{4}}\left(S_{\mu \nu }-\frac{1}{2}\gamma_{\mu \nu }S\right)\right]\\ \bar{R}-\sigma \left(K^{2}-K^{\mu \nu }K_{\mu \nu }\right)=-\frac{8\pi G}{c^{4}}\left(S+\sigma \kappa \right)\\ D_{\mu }K_{\nu }^{\mu }-D_{\nu }K=\frac{8\pi G}{c^{4}}p_{\nu } \end{array}$

## 5. The ADM Hamiltonian Formulation

The 4+1 ADM formalism can be put into Hamiltonian form with phase space variables $\gamma_{\mu \nu }$ and conjugate momentum $\pi_{\mu \nu }$. This formulation follows the general outline for the 3+1 formalism in standard GR, and so only the most important steps will be summarized here.

The ADM parameterization (69) splits the configuration space variable $g_{\alpha \beta }$ in the field equations into $g_{\alpha \beta }\left(\gamma_{\mu \nu },N^{\mu },N\right)$. Because *N* and $N^{\mu }$ are not dynamical, the phase space consists of $\gamma_{\mu \nu }$ and ${\dot{\gamma }}_{\mu \nu }=\partial_{5}\gamma_{\mu \nu }$, where from (144) we write

(173) $${\dot{\gamma }}_{\mu \nu }=\frac{1}{c_{5}}L_{\tau }\;\gamma_{\mu \nu }=L_{N}\;\gamma_{\mu \nu }+2NK_{\mu \nu }$$

with the sign in the definition of $K_{\mu \nu }$ reversed by convention. Contracting on α and β in (163) and combining with the scalar Gauss relation (153) leads to

(174) $$R=\bar{R}-\sigma \left(K^{2}-K^{\alpha \beta }K_{\alpha \beta }\right)+2\sigma \nabla_{\alpha }\left(n^{\beta }\nabla_{\beta }n^{\alpha }-n^{\alpha }\nabla_{\beta }n^{\beta }\right)$$

so that discarding the total gradient, the Einstein–Hilbert action for GR in the absence of matter becomes

(175) $$S_{ADM}\left[\gamma_{\mu \nu },{\dot{\gamma }}_{\mu \nu },N^{\mu },N\right]=\int d\tau d^{4}x\;\sqrt{\gamma }N\left[\bar{R}-\sigma \left(K^{\mu \nu }K_{\mu \nu }-K^{2}\right)\right]$$

where $\gamma =\left|\det\gamma_{\mu \nu }\right|$. The DeWitt metric is defined as

(176) $$G^{\mu \nu \lambda \rho }=\frac{1}{2}\left(\gamma^{\mu \lambda }\gamma^{\nu \rho }+\gamma^{\mu \rho }\gamma^{\nu \lambda }-2\gamma^{\mu \nu }\gamma^{\lambda \rho }\right)$$

with inverse in *D* dimensions

(177) $$G_{\mu \nu \lambda \rho }=\frac{1}{2}\left(\gamma_{\lambda \zeta }\gamma_{\rho \kappa }+\gamma_{\lambda \kappa }\gamma_{\rho \zeta }-\frac{2}{D-1}\gamma_{\lambda \rho }\gamma_{\zeta \kappa }\right)$$

in terms of which

(178) $$G^{\mu \nu \lambda \rho }K_{\mu \nu }K_{\lambda \rho }=\frac{1}{2}\left(\gamma^{\mu \lambda }\gamma^{\nu \rho }+\gamma^{\mu \rho }\gamma^{\nu \lambda }-2\gamma^{\mu \nu }\gamma^{\lambda \rho }\right)K_{\mu \nu }K_{\lambda \rho }=K^{\mu \nu }K_{\mu \nu }-K^{2}$$

so that

(179) $$L_{ADM}\left[\gamma_{\mu \nu },{\dot{\gamma }}_{\mu \nu },N^{\mu },N\right]=\sqrt{\gamma }N\left[-\sigma G^{\mu \nu \lambda \rho }K_{\mu \nu }K_{\lambda \rho }+\bar{R}\right].$$

Because $K^{\mu \nu }$ is first-order in derivatives, the first term has the form of kinetic energy. The canonical conjugate momentum to $\gamma_{\mu \nu }$ is

(180) $$\pi^{\mu \nu }=\frac{\partial L_{ADM}}{\partial {\dot{\gamma }}_{\mu \nu }}=-2\sigma \sqrt{\gamma }\;N\;G^{\zeta \kappa \lambda \rho }\;K_{\lambda \rho }\;\frac{\partial K_{\zeta \kappa }}{\partial {\dot{\gamma }}_{\mu \nu }}$$

so that using (173) to obtain

(181) $$\frac{\partial K_{\zeta \kappa }}{\partial {\dot{\gamma }}_{\mu \nu }}=\frac{1}{2N}\delta_{\zeta }^{\mu }\delta_{\kappa }^{\nu }$$

we find

(182) $$\pi^{\mu \nu }=-\sigma \sqrt{\gamma }\left(K^{\mu \nu }-\gamma^{\mu \nu }K\right)$$

with trace

(183) $$\pi =\gamma_{\mu \nu }\pi^{\mu \nu }=\sigma \left(D-1\right)\sqrt{\gamma }K\;\;⟶\;\;K=\frac{\sigma }{\left(D-1\right)\sqrt{\gamma }}\pi .$$

Writing $K^{\mu \nu }$ in terms of $\pi^{\mu \nu }$

(184) $$K^{\mu \nu }=-\frac{\sigma }{\sqrt{\gamma }}\left(\pi^{\mu \nu }-\gamma^{\mu \nu }\frac{1}{\left(D-1\right)}\pi \right)$$

and lowering the indices of $\pi^{\mu \nu }$

(185) $$G_{\mu \nu \lambda \rho }\pi^{\lambda \rho }=\pi_{\mu \nu }-\frac{1}{D-1}\gamma_{\mu \nu }\pi =-\sigma \sqrt{\gamma }K_{\mu \nu }$$

we see that $K_{\mu \nu }$ represents the momentum conjugate to $\gamma_{\mu \nu }$. Replacing $K_{\mu \nu }$ in (173), we can write the velocity as

(186) $${\dot{\gamma }}_{\mu \nu }=-\sigma \frac{2N}{\sqrt{\gamma }}G_{\mu \nu \lambda \rho }\pi^{\lambda \rho }+L_{N}\gamma_{\mu \nu }$$

in terms of the momentum and configuration variable. Because $\bar{R}$ is independent of the lapse *N* and shift $N^{\mu }$, the Lagrangian $L_{ADM}\left[\gamma_{\mu \nu },{\dot{\gamma }}_{\mu \nu },N^{\mu },N\right]$ contains no derivatives of $N,N^{\mu }$ and these act as Lagrange multipliers enforcing as constraints their conjugates. Thus, we find the Hamiltonian constraint from

(187) $$0=p_{N}=-\frac{\partial L_{ADM}}{\partial \dot{N}}=\sqrt{\gamma }\left[-\sigma \left(K^{\mu \nu }K_{\mu \nu }-K^{2}\right)-\bar{R}\right]=H$$

and the momentum constraint is

(188) $$0=-\frac{\partial L_{ADM}}{\partial N_{\mu }}=2\sigma \sqrt{\gamma }\left(D_{\nu }K^{\nu \mu }-D^{\mu }K\right)=H^{\mu }.$$

Using (182), we can also write

(189) $$H^{\nu }=2\sigma \sqrt{\gamma }D_{\mu }\left(G^{\mu \nu \lambda \rho }K_{\lambda \rho }\right)=\sigma 2D_{\mu }\pi^{\mu \nu }.$$

Performing the Legendre transformation to the Hamiltonian density

(190) $$\begin{array}{ccc} H_{ADM}= & & \;\pi^{\mu \nu }{\dot{\gamma }}_{\mu \nu }-L_{ADM}\left[\gamma_{\mu \nu },{\dot{\gamma }}_{\mu \nu },N^{\mu },N\right]\\ = & & \;-\sigma N\sqrt{\gamma }G^{\mu \nu \lambda \rho }K_{\mu \nu }K_{\lambda \rho }+2\pi^{\mu \nu }D_{\mu }N_{\nu }-\sqrt{\gamma }NR \end{array}$$

where the Lagrange multipliers $N,N^{\mu }$ do not require kinetic terms. Integrating by parts and discarding the total gradient provides

(191) $$2\pi^{\mu \nu }D_{\mu }N_{\nu }=2D_{\mu }\left(\pi^{\mu \nu }N_{\nu }\right)-N_{\nu }\left(2D_{\mu }\pi^{\mu \nu }\right)=N_{\nu }H^{\nu }$$

and using (187), we arrive at

(192) $$H_{ADM}=NH+N_{\nu }H^{\nu }.$$

Writing the Hamiltonian in the form

(193) $$H_{ADM}=\pi^{\mu \nu }{\dot{\gamma }}_{\mu \nu }+{\dot{p}}_{N}H+{\dot{p}}_{N^{\mu }}H^{\mu }-L_{ADM}$$

the Hamiltonian and momentum constraints H=0 and $H^{\nu }=0$ are seen to be secondary constraints arising from the requirement that the primary constraints $p_{N}=0$ and $p_{N^{\mu }}=0$ are preserved under time evolution,

(194) $${\dot{p}}_{N}=\left\{p_{N},H_{ADM}\right\}=0\;{\dot{p}}_{N^{\mu }}=\left\{p_{N^{\mu }},H_{ADM}\right\}=0.$$

The Einstein equations

(195) $$R_{\mu \nu }-\frac{1}{2}\gamma_{\mu \nu }R=0$$

then follow from

(196) $${\dot{\gamma }}_{\mu \nu }=\left\{\gamma_{\mu \nu },H_{ADM}\right\}\;\;{\dot{\pi }}^{\mu \nu }=\left\{\pi^{\mu \nu },H_{ADM}\right\}$$

for the canonical variables

(197) $$\left\{\gamma_{\mu \nu },\pi^{\lambda \rho }\right\}=\frac{1}{2}\left(\delta_{\mu }^{\lambda }\delta_{\nu }^{\rho }+\delta_{\mu }^{\rho }\delta_{\nu }^{\lambda }\right).$$

The equation for ${\dot{\gamma }}_{\mu \nu }$ simply reproduces the definition of $\pi^{\mu \nu }$, since *R* does not contain ${\dot{\gamma }}_{\mu \nu }$ and so $\left\{\gamma_{\mu \nu },R\right\}=0$. The Einstein equations are thus equivalent to ${\dot{\pi }}^{\mu \nu }$.

## 6. Weak Gravitation in the SHP Formalism

### 6.1. Weak Field Approximation

The weak field approximation poses the metric as a small perturbation of the 5D flat metric

(198) $$g_{\alpha \beta }=\eta_{\alpha \beta }+h_{\alpha \beta }\;⟶\;\partial_{\gamma }g_{\alpha \beta }=\partial_{\gamma }h_{\alpha \beta }\;\;{\left(h_{\alpha \beta }\right)}^{2}\approx 0$$

(199) $$\eta_{\alpha \beta }=\mathrm{diag}\left(-1,1,1,1,\sigma \right)$$

with inverse

(200) $$g^{\alpha \beta }=\eta^{\alpha \beta }-h^{\alpha \beta }$$

so that the 5D Ricci tensor reduces to the linear terms

(201) $$R_{\alpha \beta }\approx \frac{1}{2}\left(\partial_{\beta }\partial_{\gamma }h_{\alpha }^{\gamma }+\partial_{\alpha }\partial_{\gamma }h_{\beta }^{\gamma }-\partial^{\gamma }\partial_{\gamma }h_{\alpha \beta }-\partial_{\alpha }\partial_{\beta }h\right).$$

Covariance of $h_{\alpha \beta }$ under a coordinate translation $x^{\prime \alpha }=x^{\alpha }+\Lambda^{\alpha }\left(x\right)$ allows us to impose the Lorenz gauge condition

(202) $$\partial^{\beta }\left(h_{\alpha \beta }-\frac{1}{2}\eta_{\alpha \beta }h\right)=0\;⟶\;\partial^{\beta }h_{\alpha \beta }=\frac{1}{2}\partial_{\alpha }h$$

putting the 5D Ricci tensor into the form

(203) $$R_{\alpha \beta }\approx -\frac{1}{2}\partial^{\gamma }\partial_{\gamma }h_{\alpha \beta }$$

and so we obtain the 5D wave equation

(204) $$-\partial^{\gamma }\partial_{\gamma }h_{\alpha \beta }=\frac{16\pi G}{c^{4}}\left(T_{\alpha \beta }-\frac{1}{2}{\hat{\eta }}_{\alpha \beta }S\right).$$

A Green’s function satisfying

(205) $$\partial^{\gamma }\partial_{\gamma }G\left(x,\tau \right)=-\delta^{4}\left(x\right)\delta \left(\tau \right)$$

provides the generic solution

(206) $$h_{\alpha \beta }=\frac{16\pi G}{c^{4}}\int d^{4}x\;d\tau \;G\left(x-x^{\prime },\tau -\tau^{\prime }\right)\left(T_{\alpha \beta }\left(x^{\prime },\tau^{\prime }\right)-\frac{1}{2}{\hat{\eta }}_{\alpha \beta }S\left(x^{\prime },\tau^{\prime }\right)\right).$$

Introducing the notation

(207) $$\xi^{\alpha }\left(\tau \right)=\frac{1}{c}u^{\alpha }\left(\tau \right)=\frac{1}{c}\frac{dx^{\alpha }}{d\tau }$$

the mass–energy–momentum tensor (42) is

(208) $$T^{\alpha \beta }=m\rho \left(x,\tau \right){\dot{x}}^{\alpha }{\dot{x}}^{\beta }=m\rho \left(x,\tau \right)u^{\alpha }u^{\beta }=mc^{2}\rho \left(x,\tau \right)\xi^{\alpha }\xi^{\beta }$$

for some spacetime event density $\rho \left(x,\tau \right)$. Denoting

(209) $$G\left[\rho (x,\tau )\right]=\int d^{4}x\;d\tau \;G\left(x-x^{\prime },\tau -\tau^{\prime }\right)\rho \left(x^{\prime },\tau^{\prime }\right)$$

we have

(210) $$h_{\alpha \beta }=\frac{16\pi Gm}{c^{2}}G\left[\rho (x,\tau )[-]\right]\left(\xi_{\alpha }\xi_{\beta }-\frac{1}{2}{\hat{\eta }}_{\alpha \beta }\;{\hat{\xi }}^{2}\right)$$

where ${\hat{\xi }}^{2}={\hat{\eta }}^{\alpha \beta }\xi_{\alpha }\xi_{\beta }$. Considering a source distribution evolving along the *t*-axis in its rest frame, we have

(211) $$u=\left(c,0,0,0,c_{5}\right)\;⟶\;\xi =\left(1,0,0,0,\frac{c_{5}}{c}\right)\;⟶\;{\hat{\eta }}_{\alpha \beta }\xi^{\alpha }\xi^{\beta }=-1$$

from which the perturbation becomes

(212) $$\begin{array}{ccc} h_{00}=\frac{8\pi Gm}{c^{2}}G\left[\rho (x,\tau )\right]\; & \; & h_{05}=\frac{16\pi Gm}{c^{2}}\frac{c_{5}}{c}G\left[\rho (x,\tau )\right]\\ h_{ij}=\frac{8\pi Gm}{c^{2}}G\left[\rho (x,\tau )\right]\delta_{ij} & \; & h_{55}=\frac{16\pi Gm}{c^{2}}\frac{c_{5}^{2}}{c^{2}}G\left[\rho (x,\tau )\right] \end{array}$$

where electromagnetic phenomenology [3] indicates that $c_{5}/c\ll 1$. Writing the approximate Green’s function used in electrodynamics [35]

(213) $$G\left(x,\tau \right)=\frac{1}{2\pi }\delta \left(x^{2}\right)\delta \left(\tau \right)$$

and taking a uniform density $\rho \left(x\right)=\delta^{3}\left(x\right)$ evenly spread along the *t*-axis leads to the spacetime metric

(214) $$g_{\mu \nu }=\mathrm{diag}\left[-1+\frac{2Gm}{c^{2}r},\left(1+\frac{2Gm}{c^{2}r}\right)\delta_{ij}\right]$$

which becomes the Schwarzschild metric when expressed in the isotropic spherical coordinates [36] defined through

(215) $$R=r{\left(1+\frac{GM}{2c^{2}r}\right)}^{2}.$$

It is worth noting how this result would be different if we had not broken the 5D flat metric $\eta_{\alpha \beta }\;⟶\;{\hat{\eta }}_{\alpha \beta }$ in constructing the field equations in Section 3. We would then have (86) for the field equations, which produce the perturbation

(216) $$\begin{array}{ccc} h_{00}=\frac{2}{3}\;\frac{4\pi Gm}{c^{2}}G\left[\rho (x,\tau )\right]\; & \; & h_{05}=\frac{2}{3}\;\frac{4\pi Gm}{c^{2}}\frac{c_{5}}{c}G\left[\rho (x,\tau )\right]\\ h_{ij}=\frac{1}{3}\;\frac{4\pi Gm}{c^{2}}G\left[\rho (x,\tau )\right]\delta_{ij} & \; & h_{55}=\frac{1}{3}\;\frac{4\pi Gm}{c^{2}}\frac{c_{5}^{2}}{c^{2}}G\left[\rho (x,\tau )\right] \end{array}$$

leading to the phenomenologically unacceptable result $h_{00}=2h_{ij}$. This shows that preserving the O(3,1) covariance of the mass terms is not only physically reasonable, but actually required for a reasonable theory.

### 6.2. The 4+1 Decomposition for the Linearized Theory

Discarding terms of order ${\left(h_{\alpha \beta }\right)}^{2}\approx 0$, the ADM decomposition allows us to identify the terms of the perturbed metric as

(217) $$∥g_{\alpha \beta }∥=\left[\begin{array}{cc} \gamma_{\mu \nu } & N_{\mu }\\ N_{\mu } & \sigma N^{2}+\gamma_{\mu \nu }N^{\mu }N^{\nu } \end{array}\right]=\left[\begin{array}{cc} \eta_{\mu \nu }+h_{\mu \nu } & h_{\mu 5}\\ h_{\mu 5} & \eta_{55}+h_{55} \end{array}\right]$$

from which

(218) $$\sigma N^{2}+\gamma_{\mu \nu }N^{\mu }N^{\nu }\approx \sigma N^{2}=\sigma +h_{55}\;⟶\;N=\sqrt{1+\sigma h_{55}}\approx 1+\frac{1}{2}\sigma h_{55}$$

and the unit normal is

(219) $$\begin{array}{ccc} n_{\alpha }= & & \;\sigma N\delta_{\alpha }^{5}=\sigma \sqrt{1+\sigma h_{55}}\delta_{\alpha }^{5}=\sigma \left(1+\frac{1}{2}\sigma h_{55}\right)\delta_{\alpha }^{5} \end{array}$$

(220) $$\begin{array}{ccc} n^{\alpha }= & & -h_{5}^{\mu }\delta_{\mu }^{\alpha }+\left(1-\frac{1}{2}\sigma h_{55}\right)\delta_{5}^{\alpha }. \end{array}$$

Inserting these into (125) and discarding terms of the type $\Gamma_{\alpha \beta }^{\gamma }\times h_{\alpha^{\prime }\beta^{\prime }}\approx 0$, we may calculate the extrinsic curvature directly as

(221) $$K_{\alpha \beta }=\frac{1}{2}\partial_{\alpha }h_{55}\delta_{\beta }^{5}+\sigma \Gamma_{\alpha \beta }^{5}+\delta_{\alpha }^{5}\left(\frac{1}{2}\partial_{5}h_{55}\delta_{\beta }^{5}+\sigma \Gamma_{5\beta }^{5}\right)$$

which using $\delta_{\nu }^{5}=0$ provides

(222) $$K_{\mu \nu }=\Gamma_{5\mu \nu }$$

showing explicitly that the extrinsic curvature recovers the 5-components of the 5D connection not present in the 4D intrinsic curvature. By definition

(223) $$\Gamma_{5\mu \nu }=\frac{1}{2}\left(\partial_{\nu }h_{5\mu }+\partial_{\mu }h_{5\nu }-\partial_{5}h_{\mu \nu }\right)$$

and so (222) shows that $K_{\mu \nu }\times h_{\alpha \beta }\approx 0$. Discarding all terms of order ${\left(h_{\alpha \beta }\right)}^{2}\approx 0$, the initial value problem reduces to

(224) $$\begin{array}{c} \frac{1}{c_{5}}\partial_{\tau }\gamma_{\mu \nu }=-2K_{\mu \nu }+\partial_{\mu }h_{5\nu }+\partial_{\nu }h_{5\mu }\\ \frac{1}{c_{5}}\partial_{\tau }K_{\mu \nu }=\frac{1}{2}\partial_{\mu }\partial_{\nu }h_{55}-\sigma \left[{\bar{R}}_{\mu \nu }-\frac{8\pi G}{c^{4}}\left(S_{\mu \nu }-\frac{1}{2}\eta_{\mu \nu }S\right)\right]\\ \bar{R}=-\frac{8\pi G}{c^{4}}\left(S+\sigma \kappa \right)\\ \partial_{\mu }K_{\nu }^{\mu }-\partial_{\nu }K=\frac{8\pi G}{c^{4}}p_{\nu }. \end{array}$$

Writing $\partial_{5}=\left(1/c_{5}\right)\partial_{\tau }$ in (223) we see the the evolution equation for $\gamma_{\mu \nu }$ simply restates (222).

### 6.3. Insights from the Linearized Theory

In the linearized theory, the initial value problem can be derived directly from the definition of the 5D Ricci tensor

(225) $$R_{\alpha \beta }\approx \frac{1}{2}\left(\partial_{\beta }\partial^{\gamma }h_{\gamma \alpha }+\partial_{\alpha }\partial^{\gamma }h_{\gamma \beta }-\partial_{\gamma }\partial^{\gamma }h_{\alpha \beta }-\partial_{\alpha }\partial_{\beta }h\right).$$

We expand the spacetime components as

(226) $$R_{\mu \nu }=R_{\mu \nu }^{\left(4\right)}+\frac{1}{2}\left(\partial_{\nu }\partial^{5}h_{5\mu }+\partial_{\mu }\partial^{5}h_{5\nu }-\partial_{5}\partial^{5}h_{\mu \nu }-\sigma \partial_{\mu }\partial_{\nu }h_{55}\right)$$

where $R_{\mu \nu }^{\left(4\right)}$ is the standard 4D intrinsic curvature. Explicit calculation of the projected Ricci tensor using the contracted Gauss relation (152) with the unit normals (219) and (220) shows that in weak gravitation ${\bar{R}}_{\mu \nu }=R_{\mu \nu }^{\left(4\right)}$. Using (222) and (223) in (226) we obtain

(227) $$R_{\mu \nu }={\bar{R}}_{\mu \nu }+\sigma \partial_{5}K_{\mu \nu }-\frac{1}{2}\sigma \partial_{\mu }\partial_{\nu }h_{55}$$

and writing the field Equation (99) for $R_{\mu \nu }$ this becomes

(228) $$\frac{1}{c_{5}}\partial_{\tau }K_{\mu \nu }=\frac{1}{2}\partial_{\mu }\partial_{\nu }h_{55}-\sigma \left[{\bar{R}}_{\mu \nu }-\frac{8\pi G}{c_{4}}\left(T_{\mu \nu }-\frac{1}{2}P_{\mu \nu }S\right)\right]$$

which recovers the evolution equation for $K_{\mu \nu }$ in (224). Thus, as expected, the evolution equations, representing the dynamical degrees of freedom, follow from the spacetime components of the 5D field equations along with the definition of $K_{\mu \nu }$.

Imposing the Lorenz gauge condition on the 5-components of the Ricci tensor

(229) $$R_{5\beta }=-\frac{1}{2}\partial_{\gamma }\partial^{\gamma }h_{5\beta }+\frac{1}{2}\left(\partial_{\beta }\partial^{\gamma }h_{\gamma 5}+\partial_{5}\partial^{\gamma }h_{\gamma \beta }-\partial_{5}\partial_{\beta }h\right)$$

where $h=\eta^{\alpha \beta }h_{\alpha \beta }$, we obtain from (99)

(230) $$R_{5\beta }=-\frac{1}{2}\partial^{\gamma }\partial_{\gamma }h_{5\beta }=\frac{8\pi G}{c^{4}}T_{5\beta }$$

and neglecting $T_{\alpha \beta }\times h_{\alpha^{\prime }\beta^{\prime }}\approx 0$ we can write the sources as

(231) $$\kappa =n_{\alpha }n_{\beta }T^{\alpha \beta }≃T^{55}\;\;p_{\mu }=-n_{\alpha }P_{\mu \mu^{\prime }}T^{\alpha \mu^{\prime }}≃-\sigma T_{\mu }^{5}$$

satisfying wave equations

(232) $$\frac{1}{2}\partial^{\gamma }\partial_{\gamma }h_{5\mu }=\frac{8\pi G}{c^{4}}p_{\mu }\;\;\frac{1}{2}\partial^{\gamma }\partial_{\gamma }h_{55}=-\frac{8\pi G}{c^{4}}\kappa .$$

Taking the trace of (228) with the induced metric $\gamma_{\mu \nu }=\eta_{\mu \nu }+h_{\mu \nu }$ and rearranging leads to

(233) $$\bar{R}=-\frac{8\pi G}{c^{4}}S-\sigma \partial_{5}K-\frac{1}{2}\partial^{\mu }\partial_{\mu }h^{55}.$$

We may evaluate the trace of $K_{\mu \nu }$ using (222) along with the Lorenz gauge condition in the form $\partial^{\beta }h_{\alpha \beta }=\frac{1}{2}\partial_{\alpha }h$ to find

(234) $$K=-\frac{1}{2}\sigma \partial_{5}h^{55}=-\frac{1}{2}\partial^{5}h^{55}$$

so that (233) becomes

(235) $$\bar{R}=-\frac{8\pi G}{c^{4}}S+\frac{1}{2}\sigma \partial_{5}\partial^{5}h^{55}+\frac{1}{2}\sigma \partial^{\mu }\partial_{\mu }h^{55}=-\frac{8\pi G}{c^{4}}S+\frac{1}{2}\sigma \partial^{\gamma }\partial_{\gamma }h^{55}.$$

Now inserting the second of (232) we obtain

(236) $$\bar{R}=-\frac{8\pi G}{c^{4}}\left(S+\sigma \kappa \right)$$

which we recognize as the Hamiltonian constraint in the linearized form. Combining

(237) $$\partial_{\nu }K=-\frac{1}{2}\partial_{\nu }\partial^{5}h_{55}$$

and

(238) $$\partial_{\mu }K_{\nu }^{\mu }=\frac{1}{2}\left(-\partial_{5}\partial^{\mu }h_{\mu \nu }+\partial^{\mu }\partial_{\mu }h_{5\nu }+\frac{1}{2}\partial_{\nu }\partial_{5}h-\partial_{\nu }\partial^{5}h_{55}\right)$$

with the Lorenz gauge we obtain

(239) $$\partial_{\mu }K_{\nu }^{\mu }-\partial_{\nu }K=\frac{1}{2}\partial^{\gamma }\partial_{\gamma }h_{5\nu }=\frac{8\pi G}{c^{4}}p_{\nu }$$

which we recognize as the momentum constraint. Thus, as expected, the 5-components of the field Equation (99) provide the non-evolving the constraints.

The equilibrium condition for the initial value problem is $\partial_{\tau }\;⟶\;0$ so that

(240) $$\begin{array}{ccc} 0= & & \;-2K_{\mu \nu }+\partial_{\mu }h_{5\nu }+\partial_{\nu }h_{5\mu } \end{array}$$

(241) $$\begin{array}{ccc} 0= & & \;\frac{1}{2}\partial_{\mu }\partial_{\nu }h_{55}-\sigma \left[{\bar{R}}_{\mu \nu }-\frac{8\pi G}{c^{4}}\left(S_{\mu \nu }-\frac{1}{2}\eta_{\mu \nu }S\right)\right] \end{array}$$

(242) $$\begin{array}{ccc} \bar{R}= & & \;-\frac{8\pi G}{c^{4}}\left(S+\sigma \kappa \right) \end{array}$$

(243) $$\begin{array}{ccc} \partial_{\mu }K_{\nu }^{\mu }-\partial_{\nu }K= & & \;\frac{8\pi G}{c^{4}}p_{\nu }. \end{array}$$

Taking the trace of (241) we find

(244) $$\bar{R}=\frac{1}{2}\sigma \partial^{\mu }\partial_{\mu }h_{55}-\frac{8\pi G}{c^{4}}S=-\frac{8\pi G}{c^{4}}S-\frac{1}{2}\sigma \partial^{\mu }\partial_{\mu }h^{55}=-\frac{8\pi G}{c^{4}}\left(S+\sigma \kappa \right)$$

where we used the second of (232), so that the Hamiltonian constraint is implicit in the equation relating $R_{\mu \nu }$ to the sources. At equilibrium, (234) provides K=0 and the Lorenz condition reduces to

(245) $$\partial^{\mu }h_{\alpha \mu }=\frac{1}{2}\partial_{\alpha }\left(h_{\mu }^{\mu }+h_{5}^{5}\right)$$

so that using (240) we find

(246) $$\partial_{\mu }K_{\nu }^{\mu }-\partial_{\nu }K=\frac{1}{2}\partial^{\mu }\partial_{\mu }h_{5\nu }=\frac{8\pi G}{c^{4}}p_{\nu }$$

where we used the first equation in (232). Thus, the momentum constraint is also implicit in the equation for $K_{\mu \nu }$ and we see that the fields $h_{5\mu }$ and $K_{\mu \nu }$ decouple from the 4D metric $\gamma_{\mu \nu }$ so that the extrinsic curvature does not contribute to the metric at equilibrium.

We recognize the standard 4D equations in (241) along with a post-Einstein contribution from $h_{55}$. Writing

(247) $$\frac{1}{2}\partial_{\mu }\partial_{\nu }h_{55}={\hat{S}}_{\mu \nu }-\frac{1}{2}\eta_{\mu \nu }\hat{S}\;\;⟶\;\;\hat{S}=-\frac{1}{2}\partial^{\mu }\partial_{\mu }h_{55}=\frac{8\pi G}{c^{4}}\kappa$$

where we used the second equation in (232) in equilibrium, we note that the wave equation for $h_{55}$ possesses an unambiguous solution through the Green’s function for the 4D wave equation. In this form, the equilibrium field equations become

(248) $${\bar{R}}_{\mu \nu }=\frac{8\pi G}{c^{4}}\left[\left(S_{\mu \nu }-\frac{1}{2}\eta_{\mu \nu }S\right)+\sigma \left({\hat{S}}_{\mu \nu }-\frac{1}{2}\eta_{\mu \nu }\hat{S}\right)\right]$$

in which the contribution from $h_{55}$ now appears as an additional source term associated with a background scalar mass density that could play the role of ‘dark matter’ or ‘dark energy’.

## 7. Kaluza–Klein in SHP Formalism

In 1919 and 1926 Theodor Kaluza [26] and Oscar Klein [27] proposed a unification of gravitation and electromagnetism by extending Einstein’s general relativity (GR) to five dimensions. They introduced a 5D manifold with line element

(249) $$ds^{2}=g_{\alpha \beta }dx^{\alpha }dx^{\beta }\;\;x^{\alpha }=x^{0},x^{1},x^{2},x^{3},x^{5}$$

and decomposed the metric as

(250) $$g_{\alpha \beta }=\left[\begin{array}{cc} \gamma_{\mu \nu }+\phi^{2}A_{\mu }A_{\nu } & \phi^{2}A_{\mu }\\ \phi^{2}A_{\nu } & \phi^{2} \end{array}\right]\;\;g^{\alpha \beta }=\left[\begin{array}{cc} \gamma^{\mu \nu } & -A^{\mu }\\ -A^{\nu } & \phi^{-2}+A_{\rho }A^{\rho } \end{array}\right]$$

with index convention α,β=0,1,2,3,5, and μ,ν=0,1,2,3. They showed that the Einstein equations extended to 5D

(251) $$R_{\alpha \beta }-\frac{1}{2}g_{\alpha \beta }R=0$$

decompose into standard 4D gravitation and Maxwell electromagnetism, expressed through the Einstein–Hilbert–Maxwell action

(252) $$S=\int d^{4}x\;dx^{5}\;\sqrt{-g}\left(\bar{R}+\frac{1}{4}F^{\mu \nu }F_{\mu \nu }\right)$$

where

(253) $$\bar{R}=g^{\mu \nu }R_{\mu \nu }$$

is the 4D Ricci scalar and

(254) $$F_{\mu \nu }=\partial_{\mu }A_{\nu }-\partial_{\nu }A_{\mu }$$

is the Maxwell field strength. Kaluza and Klein found that the identification of $F_{\mu \nu }$ with the Maxwell field requires that ϕ= constant and that $\gamma_{\mu \nu }$ and $A_{\mu }$ be independent of the coordinate $x^{5}$. Moreover, the $x^{5}$ dimension is assumed to be compactified so that the action remains finite despite the $dx^{5}$ integration over $x^{5}$-independent fields.

Comparing with the ADM decomposition (72) of the 5D SHP metric we notice that taking σ=1 and making the replacements ϕ⟶1/N and $A_{\mu }\;⟶\;-N_{\mu }$, we have

(255) $${\left(g^{\mathrm{SHP}}\right)}_{\alpha \beta }={\left(g^{\mathrm{Kaluza}\text{-}\mathrm{Klein}}\right)}^{\alpha \beta }$$

suggesting a correspondence between SHP gravitation and Kaluza–Klein theory. But as we saw above, in SHP gravitation *N* and $N_{\mu }$ play the role of non-dynamical Lagrange multipliers, and so we expect that the electromagnetic field will not evolve with τ. In this section, we develop the Kaluza–Klein theory in the general SHP framework and show that it is consistent with SHP gravitation only in non-evolving τ-equilibrium.

In the pseudo-spacetime we defined in (21) a displacement dX leads to the interval $dX^{2}$, which Kaluza and Klein parameterized as

(256) $$dX^{2}=g_{\alpha \beta }dX^{\alpha }dX^{\beta }=\gamma_{\mu \nu }dX^{\mu }dX^{\nu }+\phi^{2}{\left(A_{\mu }dX^{\mu }+dX^{5}\right)}^{2}.$$

Following the notation of (8) we rewrite this interval in the more symmetric form

(257) $$dX^{2}=g_{\alpha \beta }dX^{\alpha }dX^{\beta }=\gamma_{\mu \nu }dX^{\mu }dX^{\nu }+{\left(a_{\mu }dX^{\mu }+a_{5}dX^{5}\right)}^{2}$$

so that the metric becomes

(258) $$g_{\alpha \beta }=\left[\begin{array}{cc} \gamma_{\mu \nu }+a_{\mu }a_{\nu } & a_{\mu }a_{5}\\ a_{\mu }a_{5} & a_{5}^{2} \end{array}\right]\;\;g^{\alpha \beta }=\left[\begin{array}{cc} \gamma^{\mu \nu } & -\frac{1}{a_{5}}a^{\mu }\\ -\frac{1}{a_{5}}a^{\nu } & \frac{1}{a_{5}^{2}}\left(1+a_{\mu }a^{\mu }\right) \end{array}\right]$$

with the apparent correspondence

(259) $$\sigma N^{2}=\frac{1}{a_{5}^{2}}\;\;N^{\mu }=-a_{5}a_{\mu }$$

between the Kaluza–Klein fields and the Lagrange multipliers of the ADM parameterization. As an initial assumption we take $g_{\alpha \beta }=g_{\alpha \beta }\left(x,\tau \right)$ for all components. The line element (257) suggests the action

(260) $$S=\int d\tau \;L\left(X,\dot{X},\tau \right)=\int d\tau \;\frac{1}{2}Mg_{\alpha \beta }{\dot{X}}^{\alpha }{\dot{X}}^{\beta }$$

where we parameterize by the external τ and, unlike Kaluza and Klein, make no reference to the proper time of the motion. The Euler–Lagrange equations lead to geodesic equations

(261) $$M{\ddot{X}}^{\gamma }+\Gamma_{\alpha \beta }^{\gamma }{\dot{X}}^{\alpha }{\dot{X}}^{\beta }=0$$

with the standard 5D Christoffel connection given in (27). The spacetime components of the geodesic equations expand as

(262) $$M{\ddot{X}}^{\mu }+\Gamma_{\lambda \rho }^{\mu }{\dot{X}}^{\lambda }{\dot{X}}^{\rho }+2\Gamma_{5\lambda }^{\mu }{\dot{X}}^{5}{\dot{X}}^{\lambda }+\Gamma_{55}^{\mu }{\dot{X}}_{5}^{2}=0$$

and we recall that SHP GR imposes

(263) $$X^{5}=c_{5}\tau \;⟶\;{\ddot{X}}^{5}\equiv 0$$

as an *a priori* constraint. But here we follow Kaluza and Klein in writing the Euler–Lagrange equation for γ=5 explicitly to obtain

(264) $$\left(\frac{d}{d\tau }\frac{\partial }{\partial {\dot{X}}^{5}}-\frac{\partial }{\partial X^{5}}\right)L=\frac{d}{d\tau }\left(a_{\mu }a_{5}{\dot{X}}^{\mu }+a_{5}^{2}{\dot{X}}^{5}\right)-\frac{1}{2}\left(\partial_{5}g_{\alpha \beta }\right){\dot{X}}^{\alpha }{\dot{X}}^{\beta }=0$$

which we will use below. Writing the spacetime part of $g_{\alpha \beta }$ as

(265) $${\bar{\gamma }}_{\alpha \beta }=\delta_{\alpha }^{\lambda }\delta_{\beta }^{\rho }\gamma_{\lambda \rho }$$

we combine the metric components as

(266) $$g_{\alpha \beta }={\bar{\gamma }}_{\alpha \beta }+a_{\alpha }a_{\beta }$$

and readily decompose the connection into 4D metric and field terms

(267) $$\Gamma_{\alpha \beta }^{\gamma }={\bar{\Gamma }}_{\alpha \beta }^{\gamma }+\frac{1}{2}g^{\gamma \delta }\left[a_{\delta }\left(\partial_{\beta }a_{\alpha }+\partial_{\alpha }a_{\beta }\right)+a_{\alpha }f_{\beta \delta }+a_{\beta }f_{\alpha \delta }\right]$$

where

(268) $$f_{\alpha \beta }=\partial_{\alpha }a_{\beta }-\partial_{\beta }a_{\alpha }$$

and

(269) $${\bar{\Gamma }}_{\gamma \alpha \beta }=\frac{1}{2}\left[\partial_{\beta }{\bar{\gamma }}_{\alpha \gamma }+\partial_{\alpha }{\bar{\gamma }}_{\beta \gamma }-\partial_{\gamma }{\bar{\gamma }}_{\alpha \beta }\right]=\frac{1}{2}\left[\delta_{\alpha }^{\lambda }\delta_{\gamma }^{\rho }\partial_{\beta }\gamma_{\lambda \rho }+\delta_{\beta }^{\lambda }\delta_{\gamma }^{\rho }\partial_{\alpha }\gamma_{\lambda \rho }-\delta_{\alpha }^{\lambda }\delta_{\beta }^{\rho }\partial_{\gamma }\gamma_{\lambda \rho }\right]$$

depends only on derivatives of the spacetime metric $\gamma_{\lambda \rho }$. The spacetime components of ${\bar{\Gamma }}_{\alpha \beta }^{\gamma }$ that enter the equations of motion (262) are

(270) $${\bar{\Gamma }}_{\alpha \beta }^{\mu }=\frac{1}{2}g^{\mu \gamma }\left[\delta_{\alpha }^{\lambda }\delta_{\gamma }^{\rho }\partial_{\beta }\gamma_{\lambda \rho }+\delta_{\beta }^{\lambda }\delta_{\gamma }^{\rho }\partial_{\alpha }\gamma_{\lambda \rho }-\delta_{\alpha }^{\lambda }\delta_{\beta }^{\rho }\partial_{\gamma }\gamma_{\lambda \rho }\right]$$

which we split into

(271) $${\bar{\Gamma }}_{\lambda \rho }^{\mu }=\Gamma_{\;\;\lambda \rho }^{(4)\mu }-\frac{1}{2}g^{\mu 5}\partial_{5}\gamma_{\lambda \rho }=\Gamma_{\;\;\lambda \rho }^{(4)\mu }-\frac{1}{2}a^{\mu }a^{5}\partial_{5}\gamma_{\lambda \rho }$$

where $\Gamma_{\;\;\lambda \rho }^{(4)\mu }$ is the standard 4D Christoffel symbol,

(272) $${\bar{\Gamma }}_{\nu 5}^{\mu }=\frac{1}{2}g^{\mu \gamma }\left[\delta_{\gamma }^{\rho }\partial_{5}\gamma_{\nu \rho }+\delta_{5}^{\lambda }\delta_{\gamma }^{\rho }\partial_{\nu }\gamma_{\lambda \rho }-\delta_{\nu }^{\lambda }\delta_{5}^{\rho }\partial_{\gamma }\gamma_{\lambda \rho }\right]=\frac{1}{2}g^{\mu \lambda }\partial_{5}\gamma_{\nu \lambda }$$

and

(273) $${\bar{\Gamma }}_{55}^{\mu }=\frac{1}{2}g^{\mu \gamma }\left[\delta_{5}^{\lambda }\delta_{\gamma }^{\rho }\partial_{5}\gamma_{\lambda \rho }+\delta_{5}^{\lambda }\delta_{\gamma }^{\rho }\partial_{5}\gamma_{\lambda \rho }-\delta_{5}^{\lambda }\delta_{5}^{\rho }\partial_{\gamma }\gamma_{\lambda \rho }\right]=0.$$

Combining with the field parts in (267) we obtain

(274) $$\Gamma_{\lambda 5}^{\mu }=\frac{1}{2}g^{\mu \lambda }\partial_{5}\gamma_{\nu \lambda }+\frac{1}{2}\left[a^{\mu }\left(\partial_{5}a_{\lambda }+\partial_{\lambda }a_{5}\right)+a_{\lambda }f_{5}^{\mu }+a_{5}f_{\lambda }^{\mu }\right]$$

(275) $$\Gamma_{55}^{\mu }={\bar{\Gamma }}_{55}^{\mu }+\frac{1}{2}g^{\mu \delta }\left[a_{\delta }\left(\partial_{5}a_{5}+\partial_{5}a_{5}\right)+a_{5}f_{5\delta }+a_{5}f_{5\delta }\right]=a^{\mu }\partial_{5}a_{5}+a_{5}f_{5}^{\mu }.$$

Considering the contribution of the 5-components to the geodesic equations, we have

(276) $$\begin{array}{cc} 2c_{5}\Gamma_{5\lambda }^{\mu }{\dot{x}}^{\lambda }+c_{5}^{5}\Gamma_{55}^{\mu } & =\;\;\left(\gamma^{\mu \lambda }+a^{\mu }a^{\lambda }\right){\dot{x}}^{\lambda }\partial_{\tau }\gamma_{\nu \lambda }\\ \; & \;\;+2c_{5}\left[a^{\mu }\partial_{\lambda }a_{5}+\frac{1}{2}\left(a^{\mu }f_{5\lambda }+a_{\lambda }f_{5}^{\;\;\mu }+a_{5}f_{\lambda }^{\;\;\mu }\right)\right]{\dot{x}}^{\lambda }\\ \; & \;\;+c_{5}^{5}\left(a^{\mu }\partial_{5}a_{5}+a_{5}f_{5}^{\;\;\mu }\right) \end{array}$$

and it is clear by inspection that the RHS will not be invariant under electromagnetic gauge transformations $a_{\alpha }\;⟶\;a_{\alpha }+\partial_{\alpha }\Lambda$ unless we adopt the three conditions imposed by Kaluza and Klein,

(277) $$a_{5}=\mathrm{constant}\;\;\partial_{5}a_{\alpha }=0\;⟶\;f_{\;5}^{\mu }=\partial_{5}a^{\mu }-\partial^{\mu }a_{5}=0\;\;\;\partial_{5}\gamma_{\nu \lambda }=0$$

which from (271) provides ${\bar{\Gamma }}_{\lambda \rho }^{\mu }\;⟶\;\Gamma_{\;\;\lambda \rho }^{(4)\mu }$. Under the Kaluza–Klein conditions, the fields behave as standard Maxwell potentials $A_{\mu }\left(x\right)$, rather than the SHP fields $a_{\alpha }\left(x,\tau \right)$, and so we change notation as

(278) $$a_{\mu }\;⟶\;A_{\mu }\;\;f_{\mu \nu }\;⟶\;F_{\mu \nu }.$$

Now (276) simplifies to

(279) $$2c_{5}\Gamma_{5\lambda }^{\mu }{\dot{x}}^{\lambda }+c_{5}^{5}\Gamma_{55}^{\mu }=c_{5}a_{5}F_{\lambda }^{\;\;\mu }{\dot{x}}^{\lambda }$$

where $a_{5}=$ constant is no longer a gauge field, and the remaining Christoffel symbols are

(280) $$\Gamma_{\lambda \rho }^{\mu }={\bar{\Gamma }}_{\lambda \rho }^{\mu }+\frac{1}{2}\gamma^{\mu \sigma }\left(a_{\lambda }F_{\rho \sigma }+a_{\rho }F_{\lambda \sigma }\right)=\Gamma_{\;\;\lambda \rho }^{(4)\mu }+\frac{1}{2}\left(a_{\lambda }F_{\rho }^{\;\;\mu }+a_{\rho }F_{\lambda }^{\;\;\mu }\right).$$

Combining (279) and (280) with (262) the geodesic equations take the form

(281) $$\begin{array}{ccc} 0 & = & M{\ddot{x}}^{\mu }+\left[\Gamma_{\;\;\lambda \rho }^{(4)\mu }+\frac{1}{2}\left(A_{\lambda }F_{\rho }^{\;\;\mu }+A_{\rho }F_{\lambda }^{\;\;\mu }\right)\right]{\dot{x}}^{\lambda }{\dot{x}}^{\rho }+c_{5}a_{5}F_{\lambda }^{\;\;\mu }{\dot{x}}^{\lambda } \end{array}$$

(282) $$\begin{array}{ccc} & = & M{\ddot{x}}^{\mu }+\Gamma_{\;\;\lambda \rho }^{(4)\mu }{\dot{x}}^{\lambda }{\dot{x}}^{\rho }+\left(A_{\lambda }{\dot{x}}^{\lambda }+c_{5}a_{5}\right)F_{\rho }^{\;\;\mu }{\dot{x}}^{\rho } \end{array}$$

where we used the index symmetry of ${\dot{x}}^{\lambda }{\dot{x}}^{\rho }$. Since now $\partial_{5}g_{\alpha \beta }=0$, we see that the basis vector in the 5-direction is a Killing vector, so that the momentum $P_{5}$ conjugate to $X^{5}$ is a constant. This is expressed in the fifth Euler–Lagrange Equation (264) as

(283) $$\frac{d}{d\tau }\frac{\partial L}{\partial {\dot{X}}^{5}}=\frac{dP_{5}}{d\tau }=\frac{d}{d\tau }\left(a_{\mu }a_{5}{\dot{X}}^{\mu }+a_{5}^{2}{\dot{X}}^{5}\right)=0,$$

imposing the condition

(284) $$A_{\mu }{\dot{x}}^{\mu }+a_{5}c_{5}=\mathrm{constant}.$$

Taking this constant to be e/c puts (282) into the form

(285) $$M{\ddot{x}}^{\mu }+\Gamma_{\;\;\lambda \rho }^{(4)\mu }{\dot{x}}^{\lambda }{\dot{x}}^{\rho }=\frac{e}{c}F_{\;\rho }^{\mu }{\dot{x}}^{\rho }$$

which we recognize as the standard Lorentz force in curved 4D spacetime.

Although we began by assuming that all components of $g_{\alpha \beta }$ may be τ-dependent as in SHP theory, we found that identification of the geodesic equations derived from the Kaluza–Klein metric as the Lorentz force associated with a gauge invariant field requires that

(286) $$a_{5}=\mathrm{constant}\;\;\partial_{5}g_{\alpha \beta }=\frac{1}{c_{5}}\partial_{\tau }\left(\gamma_{\alpha \beta }+a_{\alpha }a_{\beta }\right)=0.$$

In light of the correspondence (259) these are the equilibrium conditions under which the metric and fields undergo no τ-evolution and SHP GR reduces to standard 4D GR. Nevertheless, we see from (284) that $A_{\mu }{\dot{x}}^{\mu }=$ constant, which is not a condition associated with the standard Maxwell theory.

In the framework of SHP electrodynamics, the standard τ-independent Maxwell theory is recovered by integrating the field equations over τ as

(287) $$A_{\mu }\left(x\right)=\int d\tau \;a_{\mu }\left(x,\tau \right)\;F_{\mu \nu }\left(x\right)=\int d\tau \;f_{\mu \nu }\left(x,\tau \right)\;J_{\mu }\left(x\right)=\int d\tau \;j_{\mu }\left(x,\tau \right)$$

where $a_{\mu }\left(x,\tau \right)$ and $j_{\mu }\left(x,\tau \right)$ are the potential and current induced by an event a spacetime point *x* at time τ, while $A_{\mu }\left(x\right)$ and $J_{\mu }\left(x\right)$ are the standard Maxwell potential and current. This process, known as concatenation, is understood to sum the field contributions at a given spacetime point *x* over all times τ and describes an equilibrium state of the particles and fields.

Summarizing the connection under conditions (277) and (284) as

(288) $$\begin{array}{ccc} \Gamma_{\lambda \rho }^{\mu } & = & \frac{1}{2}\gamma^{\mu \sigma }\left(\partial_{\rho }\gamma_{\sigma \lambda }+\partial_{\lambda }\gamma_{\sigma \rho }-\partial_{\sigma }\gamma_{\lambda \rho }\right)+\frac{1}{2}\left(a_{\lambda }F_{\rho }^{\;\;\mu }+a_{\rho }F_{\lambda }^{\;\;\mu }\right) \end{array}$$

(289) $$\begin{array}{ccc} \Gamma_{\lambda 5}^{\mu } & = & a_{5}f_{\lambda }^{\;\;\mu } \end{array}$$

(290) $$\begin{array}{ccc} \Gamma_{55}^{5} & = & 0 \end{array}$$

we calculate the diagonal components of the Ricci tensor

(291) $$R_{\alpha \beta }=\partial_{\gamma }\Gamma_{\alpha \beta }^{\gamma }-\partial_{\beta }\Gamma_{\alpha \gamma }^{\gamma }+\Gamma_{\delta \gamma }^{\gamma }\Gamma_{\alpha \beta }^{\delta }-\Gamma_{\delta \beta }^{\gamma }\Gamma_{\alpha \gamma }^{\delta }$$

to find

$$\begin{array}{cc} R_{\mu \nu }= & R_{\mu \nu }^{\left(4\right)}-\frac{1}{2}\left(A_{\mu }\nabla_{\lambda }F_{\;\nu }^{\lambda }+A_{\nu }\nabla_{\lambda }F_{\;\mu }^{\lambda }\right)+\frac{1}{4}\left(F_{\;\nu }^{\lambda }F_{\lambda \mu }+F_{\;\mu }^{\lambda }F_{\lambda \nu }\right) \end{array}$$

(292) $$\begin{array}{ccc} & & -\frac{1}{4}A_{\mu }A_{\nu }F_{\lambda \rho }F^{\lambda \rho } \end{array}$$

(293) $$\begin{array}{ccc} R_{55} & = & \frac{1}{4}F_{\lambda \rho }F^{\lambda \rho } \end{array}$$

where $R_{\mu \nu }^{\left(4\right)}$ is the standard Ricci tensor in 4D, and the Ricci scalar is

(294) $$R=R^{\left(4\right)}+\frac{1}{4}F_{\lambda \rho }F^{\lambda \rho }.$$

The Einstein–Hilbert action is then

(295) $$S=\int d^{5}X\sqrt{-g}R=\int d^{5}X\sqrt{-g}\left(R^{\left(4\right)}+\frac{1}{4}F_{\lambda \rho }F^{\lambda \rho }\right)$$

taking the form of 4D gravitation with the electromagnetic field as its source. Since the integrand is independent of $X^{5}$, it is here that Kaluza and Klein require that this fifth dimension must be compactified. As we saw in (276), permitting the metric $g_{\alpha \beta }$ to depend on τ introduces a variety of new non-gauge-invariant terms to $\Gamma_{\alpha \beta }^{\gamma }$. These terms would also appear in the Ricci tensor, disrupting identification of the action with the 4D Einstein–Hilbert action.

## 8. Discussion

As seen in Section 6, we can obtain a solution to the SHP field equations in weak gravitation that is recognizable as the Schwarzschild metric (214) by considering a source distribution evolving along the *t*-axis in its rest frame. This result, which is at equilibrium in τ and does not evolve, is induced by a static matter density $\rho \left(x\right)=\delta^{3}\left(x\right)$ describing matter evenly spread along the *t*-axis. We may understand this density as the concatenation of a time-local distribution of the type described in (287)

(296) $$\rho \left(x\right)=\int d\tau \;\rho \left(x,\tau \right)=\int d\tau \;\delta \left(t-\tau \right)\delta^{3}\left(x\right)$$

where ρ(x,τ) describes an event in its rest frame evolving uniformly along the *t*-axis. In SHP electrodynamics, it is useful to model a source particle as an ensemble of events [37] located at some point x in space and narrowly distributed along the time axis according to some probability distribution φ(t−τ), where t(τ) is the event’s nominal time coordinate and φ(s) has its maximum at φ(0). Using the lowest order approximation to the Green’s function for the 5D wave equation with

(297) $$\rho \left(x,\tau \right)=\varphi \left(t-\tau \right)\delta^{3}\left(x\right)$$

this model leads to a Coulomb-type potential

(298) $$a_{0}\left(x,\tau \right)=-\frac{\varphi \left(t-R/c-\tau \right)}{R}+o\left(\frac{1}{R^{2}}\right).$$

A test event at some spacetime point $x=(ct,R\;\hat{x})$ will experience a potential whose support is centered around the chronological time τ=t−R/c, the retarded time of the source. An observer located at this static point *x* will see the potential rise and fall in strength as the chronological time τ approaches and then retreats from the retarded time.

While this model has some intuitive appeal and is generally adequate in electrodynamics, it fails as a generalization of Newtonian gravitation. Writing the perturbation to the flat metric as

(299) $$h_{\mu \nu }=\frac{2Gm}{c^{2}R}\varphi \left(t-R/c-\tau \right)\delta_{\mu \nu }$$

the geodesic equations for a nonrelativistic test event lead to the conserved angular momentum is $L=MR^{2}\dot{\phi }$ and the radial equation

(300) $$\ddot{R}=-\frac{GM}{R^{2}}\left(\varphi -R\;\partial_{R}\varphi \right)+\frac{L^{2}}{M^{2}R^{3}}.$$

For a test event similarly evolving along its *t*-axis at a distance *R* and on the lightcone of the source at t=τ−R/c, we have φ=1, so that $g_{\mu \nu }=\eta_{\mu \nu }+h_{\mu \nu }$ recovers Newtonian gravitation. However, under the resulting gravitational force, the distance *R* will decrease, so that φ will deviate from its maximum value. Given a narrow distribution, close to but away from its maximum, the term $R\partial_{R}\varphi$ becomes large at large *R* and will generally change the sign of the force term in (300). We must therefore reject separable perturbations of type (299).

### Ansatz Metric

As a first step in an alternative direction, we propose an ansatz for the metric possessing certain expected properties. We can then use the 5D wave equation in the weak field approximation to derive the source that produces the ansatz metric, evaluate the Ricci tensor and extrinsic curvature, and set up the 4+1 evolution equations for general mass–energy–momentum configurations. While the ansatz metric for the derived source satisfies the evolution equations exactly, the procedure leads to alternative metrics found as perturbations under general sources. The construction of general expressions characterizing perturbed metrics is discussed in standard texts on numerical relativity, such as [38].

We propose an ansatz in the form

(301) $$g^{\alpha \beta }=H^{\alpha \beta }\Phi \left(t,R,\tau \right)$$

where $H^{\alpha \beta }$ is a constant kinematic term and $\Phi \left(t,R,\tau \right)$ is a spherically symmetric function containing the dependence on *t*, R=|x|, and τ. We specify the component structure of $H^{\alpha \beta }$ to be

(302) $$\begin{array}{c} g^{\mu \nu }=\mathrm{diag}\left(-1+H^{00}\Phi ,\left(1+H^{00}\Phi \right)\delta^{ij}\right)\\ g^{05}=2\xi^{5}H^{00}\Phi \;\;g^{i5}=0\;\;g^{55}=2\xi_{5}^{2}H^{00}\Phi \end{array}$$

as found from (212) for a source event evolving on the *t*-axis.

We would like a function Φ that recovers the 1/R-dependence of Newtonian gravitation but whose support is restricted to a neighborhood of τ for a given test particle. We consider a source in its rest frame evolving along its time axis as $x^{0}\left(\tau \right)=x^{5}$ and a test event similarly evolving along its time axis at a spatial distance *R*. This suggests the choice

(303) $$\Phi \left(t,R,\tau \right)=\frac{1}{\sqrt{R^{2}+{\left(x^{0}-x^{5}\right)}^{2}}}=\frac{1}{\sqrt{R^{2}+c^{2}{\left(t-\xi^{5}\tau \right)}^{2}}}$$

and we denote

(304) $$\rho =\sqrt{R^{2}+c^{2}{\left(t-\xi^{5}\tau \right)}^{2}}$$

for convenience. Evaluating the connection for this metric, we study the trajectory of a test event determined by the geodesic Equation (28) with the initial conditions

(305) $$R\left(0\right)=R\;\dot{R}\left(0\right)=0\;\;t\left(0\right)=0\;\dot{t}\left(0\right)=\xi^{5}$$

for which ρ⟶R and Φ takes on its maximum value. If the test event deviates from the trajectory $\left(x^{0},x\right)=\left(\xi^{5}\tau ,R\;\hat{x}\right)$, then the strength of the metric will diminish. Because $\Phi \left(t,R,\tau \right)$ has a maximum at $t=\xi^{5}\tau$ with respect to *t* but not with respect to *R*, this functional form does not suffer from the difficulties associated with the geodesic Equation (300).

Writing the derivatives

(306) $$\frac{\partial \Phi }{\partial t}=-\frac{c^{2}\left(t-\xi^{5}\tau \right)}{\rho^{3}}\;\;\frac{\partial \Phi }{\partial \tau }=\frac{\xi^{5}c^{2}\left(t-\xi^{5}\tau \right)}{\rho^{3}}\;\;\frac{\partial \Phi }{\partial x^{i}}=-\frac{x_{i}}{\rho^{3}}$$

leads to the connection

(307) $$\begin{array}{ccc} \Gamma_{00}^{\mu }= & & \;\frac{1}{2}H^{00}\frac{1}{\rho^{3}}\left(\delta^{\mu 0}c\left(t-\xi^{5}\tau \right)+\delta^{\mu k}x_{k}\right) \end{array}$$

(308) $$\begin{array}{ccc} \Gamma_{i0}^{\mu }= & & \;H^{00}\frac{1}{2}\frac{1}{\rho^{3}}\left[-\delta_{i}^{\mu }c\left(t-\xi^{5}\tau \right)+\delta^{\mu 0}x_{i}\right] \end{array}$$

(309) $$\begin{array}{ccc} \Gamma_{ij}^{\mu }= & & \;-\frac{1}{2}H^{00}\frac{1}{\rho^{3}}\left[\delta_{i}^{\mu }x_{j}+\delta_{j}^{\mu }x_{i}-\delta^{\mu k}\delta_{ij}x_{k}+\delta^{\mu 0}\delta_{ij}c\left(t-\xi^{5}\tau \right)\right] \end{array}$$

(310) $$\begin{array}{ccc} \Gamma_{50}^{\mu }= & & \;-\frac{1}{2}H^{00}\frac{1}{\rho^{3}}\left[2\sigma \xi^{5}\delta^{\mu k}x_{k}+\delta^{\mu 0}c\left(t-\xi^{5}\tau \right)\right] \end{array}$$

(311) $$\begin{array}{ccc} \Gamma_{5i}^{\mu }= & & \;-\frac{1}{2}H^{00}\frac{1}{\rho^{3}}\left(-\delta_{i}^{\mu }c\left(t-\xi^{5}\tau \right)+\xi^{5}\delta^{\mu 0}2\sigma x_{i}\right) \end{array}$$

(312) $$\begin{array}{ccc} \Gamma_{55}^{\mu }= & & \;H^{00}\frac{1}{\rho^{3}}\left[\delta^{\mu k}\xi_{5}^{2}x_{k}+\delta^{\mu 0}c_{5}\left(t-\xi^{5}\tau \right)\left(2\sigma -\xi^{5}\right)\right] \end{array}$$

and the equations of motion in time and space components

(313) $$\begin{array}{ccc} 0= & & \;\ddot{t}+H^{00}\frac{1}{\rho^{3}}\left\{\frac{1}{2}c^{2}\left(t-\xi^{5}\tau \right){\dot{t}}^{2}+x_{i}{\dot{x}}^{i}\dot{t}-\frac{1}{2}\left(t-\xi^{5}\tau \right)\delta_{ij}{\dot{x}}^{i}{\dot{x}}^{j}\right.\\ & & \;\left.-cc_{5}\left(t-\xi^{5}\tau \right)\dot{t}-2\sigma \xi_{5}^{2}x_{i}{\dot{x}}^{i}+cc_{5}\xi_{5}^{2}\left(t-\xi^{5}\tau \right)\left(2\sigma -\xi_{5}^{2}\right)\right\} \end{array}$$

(314) $$\begin{array}{ccc} 0= & & \;{\ddot{x}}^{k}+H^{00}\frac{1}{\rho^{3}}c^{2}\left\{\frac{1}{2}x^{k}{\dot{t}}^{2}-c\left(t-\xi^{5}\tau \right)\frac{{\dot{x}}^{k}}{c}\dot{t}-x_{j}\frac{{\dot{x}}^{k}}{c}\frac{{\dot{x}}^{j}}{c}+\frac{1}{2}x^{k}\frac{{\dot{x}}_{j}}{c}\frac{{\dot{x}}^{j}}{c}\right.\\ & & \;\left.-2\sigma \xi_{5}^{2}x^{k}\dot{t}+c_{5}\left(t-\xi^{5}\tau \right)\frac{{\dot{x}}^{k}}{c}+\xi_{5}^{4}x^{k}\right\}. \end{array}$$

In the neighborhood of the initial conditions, the equations of motion reduce to

(315) $$0=\ddot{t}+H^{00}\frac{x_{i}}{R^{3}}\frac{{\dot{x}}^{i}}{c}c\left(\dot{t}-2\sigma \xi_{5}^{2}\right)$$

(316) $$0={\ddot{x}}^{k}+H^{00}\frac{1}{R^{3}}c^{2}\left\{\frac{1}{2}x^{k}{\dot{t}}^{2}-x_{j}\frac{{\dot{x}}^{k}}{c}\frac{{\dot{x}}^{j}}{c}+\frac{1}{2}x^{k}\frac{{\dot{x}}_{j}}{c}\frac{{\dot{x}}^{j}}{c}-2\sigma \xi_{5}^{2}x^{k}\dot{t}+\xi_{5}^{4}x^{k}\right\}$$

so that neglecting $\dot{x}/c\ll 1$ in the nonrelativistic regime the time equation reduces to

(317) $$0=\ddot{t}\;⟶\;\dot{t}\left(\tau \right)=\xi^{5}$$

and the space equation becomes

(318) $$0={\ddot{x}}^{k}+H^{00}\frac{1}{R^{3}}c^{2}\xi_{5}^{2}\left\{\frac{1}{2}x^{k}-2\sigma \xi^{5}x^{k}+\xi_{5}^{2}x^{k}\right\}≃{\ddot{x}}^{k}+\frac{1}{2}c^{2}\xi_{5}^{2}H^{00}\frac{1}{R^{2}}{\hat{x}}^{k}$$

which recovers the form of Newtonian gravitation if we take $H^{00}=2GM/c^{2}$.

For a relativistic test event with the initial condition $t=\xi^{5}\tau$, the equations of motion become

(319) $$0=\ddot{t}+H^{00}\frac{1}{R^{2}}{\hat{x}}_{i}{\dot{x}}^{i}\dot{t}\;⟶\;c_{5}\ddot{t}≃-\frac{2GM}{R^{2}}{\hat{x}}_{i}\frac{{\dot{x}}^{i}}{c}$$

(320) $$0={\ddot{x}}^{k}+\frac{GM}{R^{2}}\left\{{\hat{x}}^{k}-\left(\frac{2{\hat{x}}_{j}{\dot{x}}^{k}-{\hat{x}}^{k}{\dot{x}}_{j}}{c}\right)\frac{{\dot{x}}^{j}}{c}\;\frac{c^{2}}{c_{5}^{2}}\right\}.$$

Since $c/c_{5}>1$ by assumption, while for non-tachyonic particles ${\dot{x}}^{k}<c$, we see here that the event trajectory may differ from standard post-Newtonian relativistic forms. Using the wave Equation (204) to calculate the source of the ansatz metric we find

(321) $$\frac{16\pi G}{c^{4}}\left(T_{\mu \nu }-\frac{1}{2}{\hat{\eta }}_{\mu \nu }S\right)=-\frac{1}{2}H^{00}\delta_{\mu \nu }\;\left(\nabla^{2}-\frac{1}{c^{2}}\frac{\partial^{2}}{\partial t^{2}}+\sigma \frac{1}{c_{5}^{2}}\frac{\partial^{2}}{\partial \tau^{2}}\right)\Phi$$

for any spacetime functional Φ. Combining the derivatives of (303)

(322) $$\nabla^{2}\Phi =-\frac{3c^{2}{\left(t-\xi^{5}\tau \right)}^{2}}{\rho^{5}}$$

(323) $$-\frac{1}{c^{2}}\frac{\partial^{2}}{\partial t^{2}}\Phi =\frac{r^{2}-2c^{2}{\left(t-\xi^{5}\tau \right)}^{2}}{\rho^{5}}$$

and

(324) $$\sigma \frac{1}{c_{5}^{2}}\frac{\partial^{2}}{\partial \tau^{2}}\Phi =-\sigma \frac{r^{2}-2c^{2}{\left(t-\xi^{5}\tau \right)}^{2}}{\rho^{5}}$$

we are led to

(325) $$\frac{16\pi G}{c^{4}}\left(T_{\mu \nu }-\frac{1}{2}{\hat{\eta }}_{\mu \nu }S\right)=-\frac{1}{2}\frac{H^{00}}{\rho^{5}}\left[\left(\sigma -1\right)R^{2}+\left(5-2\sigma \right)c^{2}{\left(t-\xi^{5}\tau \right)}^{2}\right]\delta_{\mu \nu }$$

and if we again take $H^{00}=2GM/c^{2}$, then

(326) $$T_{\mu \nu }=\frac{Mc^{2}}{4\pi \rho^{5}}\left[\left(\sigma -1\right)R^{2}+\left(5-2\sigma \right)c^{2}{\left(t-\xi^{5}\tau \right)}^{2}\right]\mathrm{diag}\left(-1,0,0,0\right)$$

where only the energy component $T_{00}$ is non-vanishing. The structure of this source is easiest to see for σ=1, in which case

(327) $$T_{\mu \nu }=3\frac{Mc^{2}}{4\pi \rho^{5}}{\left[c\left(t-\xi^{5}\tau \right)\right]}^{2}\mathrm{diag}\left(-1,0,0,0\right)$$

where $c^{2}{\left(t-\xi^{5}\tau \right)}^{2}/\rho^{5}$ has units of ${\mathrm{length}}^{-3}$, as expected for a particle density in space. Although $T_{\mu \nu }$ appears to vanish at $t=\xi^{5}\tau$, we recall that under this condition, ρ⟶R so that Φ becomes independent of *t* and τ. In this case, the wave Equation (321) from which we derive the source reduces to

(328) $$\partial^{\gamma }\partial_{\gamma }\left(\frac{1}{\rho }\right)\;⟶\;\nabla^{2}\left(\frac{1}{R}\right)=-4\pi \delta^{\left(3\right)}\left(x\right)$$

which describes a point source evenly spread along the *t*-axis. In contrast, at a small spatial distance $R\ll c(t-\xi^{5}\tau )$, we have $c^{2}{\left(t-\xi^{5}\tau \right)}^{2}/\rho^{5}\;⟶\;{\left[c^{2}\left(t-\xi^{5}\tau \right)\right]}^{-3}$, which describes a narrow particle density along the *t*-axis centered at $t=\xi^{5}\tau$. This localization at a coordinate time *t* determined by the chronological time τ expresses the desired τ-evolution. As expected, the source (327) describes a matter distribution evolving with τ, leading to the metric (302), which similarly evolves with τ, and the geodesic Equations (313) and (314), whose coefficients evolve with τ.

The projected Ricci tensor is found using (227)

(329) $${\bar{R}}_{\mu \nu }=H^{00}\left[-\frac{1}{2}\delta_{\mu \nu }\left(\nabla^{2}-\frac{1}{c^{2}}\frac{\partial^{2}}{\partial t^{2}}\right)+\xi^{5}\frac{1}{c_{5}}\frac{\partial }{\partial \tau }\left(\delta_{\nu 0}\frac{\partial }{\partial x^{\mu }}+\delta_{\mu 0}\frac{\partial }{\partial x^{\nu }}\right)\right]\Phi$$

and the extrinsic curvature is

(330) $$K_{\mu \nu }=-H^{00}\left[\sigma \xi^{5}\left(\delta_{\nu 0}\frac{\partial }{\partial x^{\mu }}+\delta_{\mu 0}\frac{\partial }{\partial x^{\nu }}\right)+\frac{1}{2}\delta_{\mu \nu }\frac{1}{c_{5}}\frac{\partial }{\partial \tau }\right]\Phi$$

from which

(331) $$\sigma \partial_{5}K_{\mu \nu }=H^{00}\left[-\xi^{5}\frac{1}{c_{5}}\frac{\partial }{\partial \tau }\left(\delta_{\nu 0}\frac{\partial }{\partial x^{\mu }}+\delta_{\mu 0}\frac{\partial }{\partial x^{\nu }}\right)-\sigma \frac{1}{2}\delta_{\mu \nu }\frac{1}{c_{5}^{2}}\frac{\partial^{2}}{\partial \tau^{2}}\right]\Phi .$$

We see that the off-diagonal terms in ${\bar{R}}_{\mu \nu }$ and $\sigma \partial_{5}K_{\mu \nu }$ mutually cancel, leaving the source diagonal as required.

Using any suitable functional Φ(t,R,τ) to derive an ansatz metric (302), its unperturbed source (321), and initial conditions (329) and (330), the weak field evolution equations are satisfied exactly. Writing the unperturbed source as $T_{\mu \nu }^{\left(0\right)}$, the perturbed source $T_{\mu \nu }$ can be found using standard approximation methods. For example, we may write a source parameterized by λ as

(332) $$T_{\mu \nu }=T_{\mu \nu }^{\left(0\right)}+\lambda T_{\mu \nu }^{\left(1\right)}+\lambda^{2}T_{\mu \nu }^{\left(2\right)}+\cdots$$

where $\lambda =0\;⟶\;T_{\mu \nu }=T_{\mu \nu }^{\left(0\right)}$, and we seek a perturbed metric

(333) $$h_{\alpha \beta }=h_{\alpha \beta }^{\left(0\right)}+\lambda h_{\alpha \beta }^{\left(1\right)}+\lambda^{2}h_{\alpha \beta }^{\left(2\right)}+\cdots$$

up to the desired order in λ. For example, writing

(334) $$\Phi =\frac{1}{\sqrt{r^{2}+c^{2}{\left(t-\xi^{5}\tau \right)}^{2}}}\;⟶\;\frac{1}{\sqrt{R^{2}+c^{2}A\left(t,\tau \right)}}$$

where

(335) $$A\left(t,\tau \right)={\left(t-\xi^{5}\tau \right)}^{2}+\lambda \alpha \left(t,\tau \right)$$

leads to

(336) $$\Phi ≃\frac{1}{\sqrt{R^{2}+c^{2}{\left(t-\xi^{5}\tau \right)}^{2}}}-\frac{1}{2}\frac{\lambda \alpha \left(t,\tau \right)}{R^{2}+c^{2}{\left(t-\xi^{5}\tau \right)}^{2}}$$

to first order. In the linearized theory, we also have the first-order

(337) $$R_{\mu \nu }=R_{\mu \nu }^{\left(0\right)}+\lambda R_{\mu \nu }^{\left(1\right)}\;\;K_{\mu \nu }=K_{\mu \nu }^{\left(0\right)}+\lambda K_{\mu \nu }^{\left(1\right)}$$

where $R_{\mu \nu }^{\left(0\right)}$ and $K_{\mu \nu }^{\left(0\right)}$ are derived from $T_{\mu \nu }^{\left(0\right)}$. And since the evolution equations are linear and solved exactly by $h_{\mu \nu }^{\left(0\right)}$ for $T_{\mu \nu }^{\left(0\right)}$, the evolution equations for the perturbed metric reduce to the evolution equations for the perturbation itself.

(338) $$\partial_{5}h_{\mu \nu }^{\left(1\right)}=-2K_{\mu \nu }^{\left(1\right)}+\partial_{\mu }h_{5\nu }^{\left(1\right)}+\partial_{\nu }h_{5\mu }^{\left(1\right)}$$

(339) $$\partial_{5}K_{\mu \nu }^{\left(1\right)}=-\sigma \left[R_{\mu \nu }^{\left(1\right)}-\frac{8\pi G}{c^{4}}\left(S_{\mu \nu }^{\left(1\right)}-\frac{1}{2}\eta_{\mu \nu }S^{\left(1\right)}\right)\right]$$

where we may use (329) and (330) to express $R_{\mu \nu }^{\left(1\right)}$ and $K_{\mu \nu }^{\left(1\right)}$ in terms of the perturbation $\alpha \left(t,\tau \right)$. We note that in order to preserve the structure of the evolution equations as an initial value problem, we must preserve $\partial_{5}\alpha \left(t,\tau \right)$ as an independent dynamical quantity and choose the initial values for $\alpha \left(t,0\right)$ and $\partial_{5}\alpha \left(t,0\right)$.

## Data Availability

No new data were created or analyzed in this study. Data sharing is not applicable to this article.

## Abbreviations

The following abbreviations are used in this manuscript:

SHP	Stueckelberg–Horwitz–Piron formalism
ADM	Arnowitt, Deser, and Misner formalism
LHS	Left-Hand Side
RHS	Right-Hand Side
